# Multistep Photochemical Reactions of Polypyridine-Based Ruthenium Nitrosyl Complexes in Dimethylsulfoxide

**DOI:** 10.3390/molecules25092205

**Published:** 2020-05-08

**Authors:** Nataliia Marchenko, Pascal G. Lacroix, Valerii Bukhanko, Marine Tassé, Carine Duhayon, Martial Boggio-Pasqua, Isabelle Malfant

**Affiliations:** 1 LCC (Laboratoire de Chimie de Coordination), CNRS, 205, route de Narbonne, F-31077 Toulouse, France; marchenko.nataliia01@gmail.com (N.M.); valerkabu@gmail.com (V.B.); marine.tasse@lcc-toulouse.fr (M.T.); carine.duhayon@lcc-toulouse.fr (C.D.); 2 Laboratoire de Chimie et Physique Quantiques, Université Paul Sabatier (Toulouse), UMR 5626, 218 route de Narbonne, F-31077 Toulouse, France

**Keywords:** nitric oxide, DFT computations, photochemistry, ruthenium complexes

## Abstract

The photorelease of nitric oxide (NO·) has been investigated in dimethylsulfoxide (DMSO) on two compounds of formula [Ru(R-tpy)(bpy)(NO)](PF_6_)_3_, in which bpy stands for 2,2′-bipyridine and R-tpy for the 4′-*R*-2,2′:6′,2″-terpyridine with R = H and MeOPh. It is observed that both complexes are extremely sensitive to traces of water, leading to an equilibrium between [Ru(NO)] and [Ru(NO_2_)]. The photoproducts of formula [Ru(R-tpy)(bpy)(DMSO)](PF_6_)_2_ are further subjected to a photoreaction leading to a reversible linkage isomerization between the stable Ru-DMSO_(S)_ (sulfur linked) and the metastable Ru-DMSO_(O)_ (oxygen linked) species. A set of 4 [Ru(R-tpy)(bpy)(DMSO)]^2+^ complexes (R = H, MeOPh, BrPh, NO_2_Ph) is investigated to characterize the ratio and mechanism of the isomerization which is tentatively related to the difference in absorbance between the Ru-DMSO_(S)_ and Ru-DMSO_(O)_ forms. In addition, the X-ray crystal structures of [Ru(tpy)(bpy)(NO)](PF_6_)_3_ and [Ru(MeOPh-tpy)(bpy)(DMSO_(S)_)](PF_6_)_2_ are presented.

## 1. Introduction

Nitric oxide (NO·) is a radical involved in numerous biological functions, such as blood pressure regulation, stimulation of immune response or neurotransmission, which has been observed to exhibit various anticancer, antibacterial, and anti-inflammatory activities [1]. For these reasons, intense research efforts have been directed towards the design of exogenous NO· donors capable of releasing NO· locally and quantitatively [2,3]. Among potential candidates, ruthenium-nitrosyl [Ru(NO)] complexes appear especially promising due to their usually low toxicity, good chemical stability and their capability to release NO· under irradiation in the *λ* = 300–600 nm domain, exclusively [4,5,6,7,8,9,10,11,12,13]. In practice, and to overpass the frequently modest solubility of metal-nitrosyl complexes in biological media, the complexes are generally dissolved in aqueous solutions containing a maximum in volume of 0.5% of dimethylsulfoxide (DMSO) before being tested on living cells. In this context, the stability and photochemical reactivity of [Ru(NO)] species in DMSO is naturally addressed. Indeed, DMSO is a potential ligand for ruthenium complexes [14]. Furthermore, several examples of [Ru(DMSO)] species have been found to be photo-isomerizable, which could also broaden the range of applications of these versatile species [15,16,17,18,19,20].

**Figure 1:**
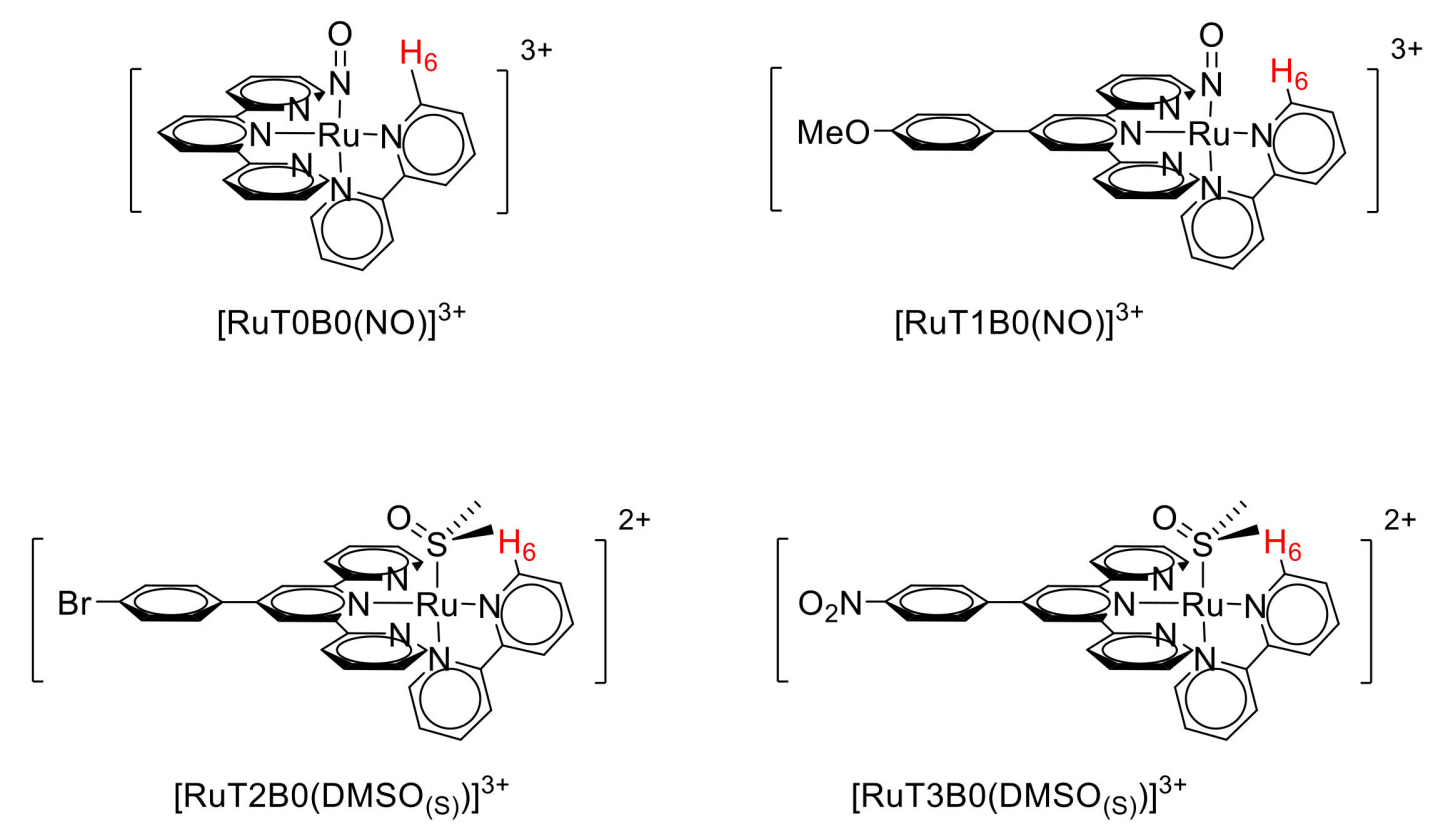
Ruthenium complexes under investigation. Proton H6 used for the NMR characterization is indicated in red color on the bipyridine ligand.

In a research effort aimed at checking out the full behavior of [Ru(NO)] complexes in DMSO environment, the chemical and photochemical properties of [Ru(MeOPh-tpy)(bpy)(NO)]^3+^ ([RuT1B0(NO)]^3+^ in Figure 1), where MeOPh-tpy is the 4′-(4-methoxyphenyl)-2,2′:6′,2″-terpyridine, and bpy the 2,2′-bipyridine, have been investigated. [RuT1B0(NO)]^3+^ has been selected as a benchmark candidate for NO· release, due to its “push-pull” character between the electron-rich methoxyphenyl and the electron-withdrawing nitrosyl (NO) substituents. This effect has long been expected to enhance the charge transfer towards the nitrosyl and hence to favor the NO· release capabilities, according to the following overall reaction [5]:

(1) $$\left[Ru\left(NO\right)\right]+solvent\;\overset{h\nu }{\to }\;\left[Ru\left(solvent\right)\right]+NO·$$

Furthermore, the electronic “push-pull” character of [RuT1B0(NO)]^3+^ is expected to enhance the two-photon absorption (TPA) properties, which is highly desirable for practical applications in the *λ* = 600–1200 nm therapeutic window under two-photon irradiation, the method of choice for drugs photodelivery in biological media [21,22]. A reference complex of formula [Ru(tpy)(bpy)(NO)]^3+^ ([RuT0B0(NO)]^3+^ in Figure 1), will be presented in parallel to [RuT1B0(NO)]^3+^, to elucidate the NO· photorelease process in DMSO medium. In a last section, two additional complexes of formula [RuT2B0(DMSO)]^3+^ and [RuT3B0(DMSO)]^3+^, where T2 an T3 state for 4′-(4-(bromo)phenyl)-2,2′:6′,2″-terpyridine and 4′-(4-(nitro)phenyl)-2,2′:6′,2″-terpyridine, respectively, will be presented to fully investigate the possibilities for Ru-DMSO_(S)_ to Ru-DMSO_(O)_ linkage isomerizations (Figure 1).

## 2. Results and Discussion

### 2.1. Photo-Chemical Behavior of [Ru(R-tpy)(bpy)(NO)]^3+^ in DMSO: the First Step

**Figure 2:**
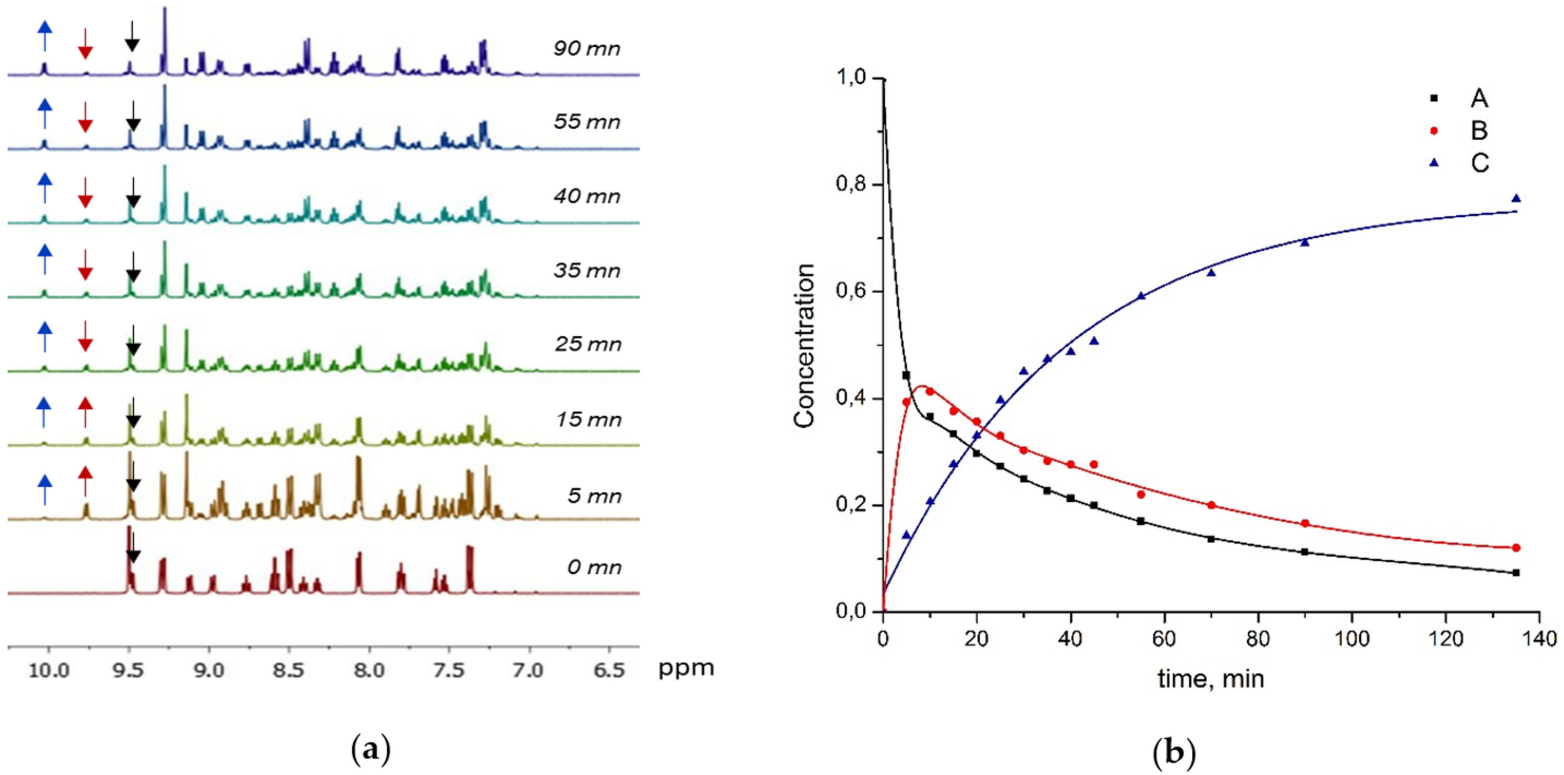
(**a**) ^1^H-NMR tracking during the irradiation of [RuT1B0(NO)](PF_6_)_3_ (A) in DMSO-*d6*, at *λ* = 395 nm (black, red, blue arrows for A, B, C respectively). (**b**) Relative concentrations of A, B, and C, from the NMR integrated signals.

The ^1^H-NMR monitoring of the continuous irradiation of [RuT1B0(NO)](PF_6_)_3_ in deuterated DMSO (DMSO-*d6*) is shown in Figure 2a. ^1^H-NMR appears to be an excellent tool for defining the coordination sphere of [Ru(tpy)(bpy)X] species. We have previously reported that the chemical shift of the proton in position 6 on the bipyridine, is very sensitive to the nature of the X ligand attached to the ruthenium center, due to the spatial proximity between H-6 and X and the subsequent different electronic environment (Figure 1) [23,24]. Three different compounds (A, B, C) are observed on the spectra, with various concentrations throughout the irradiation process. The integrations of the NMR signals allows drawing the relative concentrations in Figure 2b, where the sum of the concentrations [A] + [B] + [C] is normalized to 1, at any reaction time.

**Scheme 1:**
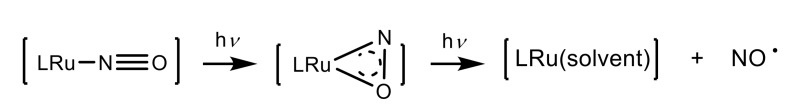
Mechanism in two steps involved in the NO· release process.

The examination of Figure 2 reveals that the starting compound (A) is gradually transformed to the final product (C), B appearing as a possible intermediate in a global photoreaction which, at first, can tentatively be written as A → B → C. This assumption has to be considered in the context of previous investigations conducted in acetonitrile, in which the irradiation of [RuT1B0(NO)](PF_6_)_3_ leads to the complete release of NO·, [RuT1B0(MeCN)](PF_6_)_2_ being clearly identified as the final photoproduct (Reaction (1)) [23,24]. Importantly, no additional or intermediate species is observed in acetonitrile, in striking contrast with the present investigation. The search for a metastable transient species in the NO· release process has become a challenging issue for experimentalists, as theoretical investigations have recently suggested that the overall reaction should necessarily imply a mechanism in two steps. Indeed, theoreticians do not consider the singlet excited state (1A*) generated after a first photon absorption as a dissociative state [25,26]. Therefore, the need of a second photon absorption would rather lead to an overall mechanism depicted in Scheme 1:

At first, it seems that the anticipated sideways-bonded isomer in Reaction (2), usually called MS2 in the literature [25,26], could be identified with B (Figure 2), which would imply a complex of formula [Ru(MeOphtpy)(bpy)(η2-NO)]^3+^, in the present case. Nevertheless, and although few examples of such MS2 states have been reported [27,28,29,30], they can hardly be stabilized at room temperature. The details of the analyses undertaken towards the full clarification of the nature and interactions of A, B, C species present in the reaction mixture will be reported as follows: (1) nature of form C; (2) nature of form B, (3) origin of form C, and (4) origin of form B.

#### 2.1.1. Nature of Form C

The final compound C can be isolated quantitatively after an irradiation of few hours (Figure 2b), and therefore is easily characterized by ^1^H-NMR spectroscopy, mass spectrometry, and X-ray diffraction analyses. The crystal structure, described in a following section, reveals that C corresponds to the expected photo-product of formula [Ru(MeOphtpy)(bpy)(DMSO_(S)_)](PF_6_)_2_, in which DMSO_(S)_ indicates that a molecule of DMSO is linked to the complex through a Ru-S bond. The NO· release can also be followed by EPR spectroscopy, as it has been already controlled in a previous investigation [23]. To further establish that C arises from the quantitative substitution of NO· by DMSO, the complex [RuT1B0(DMSO)_(S)_]^2+^ has been synthesized directly from [RuT1B0(Cl)]^+^ (see Experimental Section). It turns out from mass spectrometry and NMR spectroscopy that both chemically and photo-chemically synthesized derivatives are indeed the same species. Infrared spectroscopy provides further evidence for the quantitative replacement of NO· by DMSO in compound C, with the disappearance of the *ν*NO stretching frequency at 1940 cm^−1^ [23,24] and the concomitant appearance of the *ν*SO band at 1101 cm^−1^.

#### 2.1.2. Nature of Form B

Defining the nature of B is hampered by the fact that it appears difficult to generate this intermediate in sufficient yield and furthermore from the fact that its concentration decreases rapidly in the solution mixture (Figure 2b). To overcome this difficulty, an additional investigation has been conducted on the reference compound [RuT0B0(NO)](PF_6_)_3_, the crystal structure of which will be presented in the next section. Contrary to the situation encountered in [RuT1B0(NO)](PF_6_)_3_, the photo-generation of C appears more difficult in [RuT0B0(NO)](PF_6_)_3_, therefore B can be obtained in high yield (89%) in the reaction mixture, which favors its investigation. Mass spectrometry analyses lead to the conclusion that form B corresponds to [RuT0B0(NO_2_)](PF_6_), a species which can be generated from A, according to the following acid/base equation:

(2) $${\left[\mathrm{Ru}(\mathrm{NO})\right]}^{3+}+3\;H_{2}O\;⇌\;{\left[\mathrm{Ru}(NO_{2})\right]}^{+}+2\;H_{3}O^{+}$$

In order to further characterize B, it is interesting to note that the ^1^H-NMR chemical shifts of the bipyridine ligands are only weakly affected by the substituents present on the terpyridine moieties. Therefore, the chemical shifts related to the bipyridine fragments are assumed to be the same in [RuT0B0(NO)](PF_6_)_3_ and in [RuT1B0(NO)](PF_6_)_3_. The relevant ^1^H-NMR chemical shifts are shown in Table 1, where the different X ligands corresponds to those present during the synthetic route towards the [Ru(NO)] complexes.

The data indicate that the non-isolated form B in [RuT1B0(NO)](PF_6_)_3_ (Figure 2), and the related form B in [RuT0B0(NO)](PF_6_)_3_ correspond to the same coordination sphere. Therefore B is identified to the ruthenium complex in which NO is switched to NO_2_, in both cases.

Additional support for identifying unambiguously the nature of form B from the available ^1^H-NMR data, is provided by a computational investigation. The theoretical characterization of the A, B and C forms was performed by optimizing the geometrical structures of these species for the [RuT1B0(NO)]^3+^ complex and by computing the ^1^H-NMR chemical shifts at the H-6 position. This analysis was also carried out for other intermediate structures and photoproducts potentially produced after photoexcitation. In particular, the [Ru(MeOphtpy)(bpy)(η^2^-NO)]^3+^ intermediate (Scheme 1), the [Ru(MeOphtpy)(bpy)(ON)]^3+^ photoproduct of the linkage N → O isomerization of form A and the [Ru(MeOphtpy)(bpy)(DMSO_(O)_)]^2+^ species resulting from the linkage S → O isomerization of form C [15,16,17,18,19], were all considered. The different structures are reported in Supplementary Materials along with their respective thermal stabilities. The computed relative ^1^H-NMR chemical shifts at the H-6 position of all these species are given in Table 2 with respect to the value of this chemical shift for the initial A form. Experimentally, there is an increase of the chemical shift (deshielding) at the H-6 position on going from A to B by about 0.3 ppm (Figure 2, Table 1). This rules out the [Ru(MeOphtpy)(bpy)(η^2^-NO)]^3+^ and [Ru(MeOphtpy)(bpy)(ON)]^3+^ species for which a shielding is predicted by the computation. Moreover, a further 0.2 ppm deshielding is observed from B to C (Figure 2). This is qualitatively consistent with the variation of the chemical shift from [RuT0B0(NO_2_)]^+^ to [Ru(MeOphtpy)(bpy)(DMSO_(S)_)]^2+^, in agreement with the experimental characterizations of the B and C forms.

**Figure 3:**
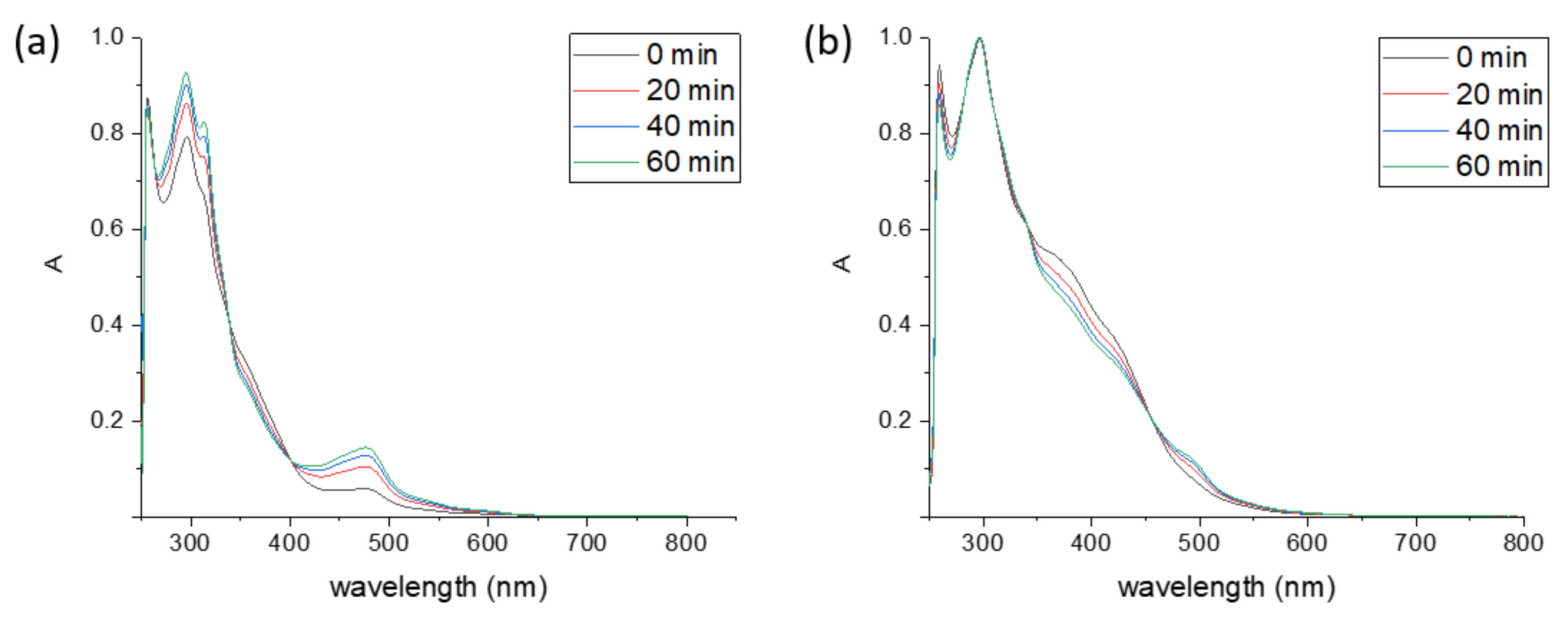
Time-evolution of UV-vis absorption spectra during 1 h without irradiation of (**a**) [RuT0B0(NO)]^3+^ and of (**b**) [RuT1B0(NO)]^3+^.

In addition, the nature of form B is also confirmed by the analysis of the UV-vis absorption spectra. The experimental spectra were recorded for freshly prepared compounds without irradiation. The spectra are shown in Figure 3 for [RuT0B0(NO)]^3+^ and [RuT1B0(NO)]^3+^. From the time evolution of these spectra in the dark, it is clear that these complexes are not thermally stable in DMSO. The presence of two isosbestic points indicates a chemical transformation from A to B without intermediate.

**Figure 4:**
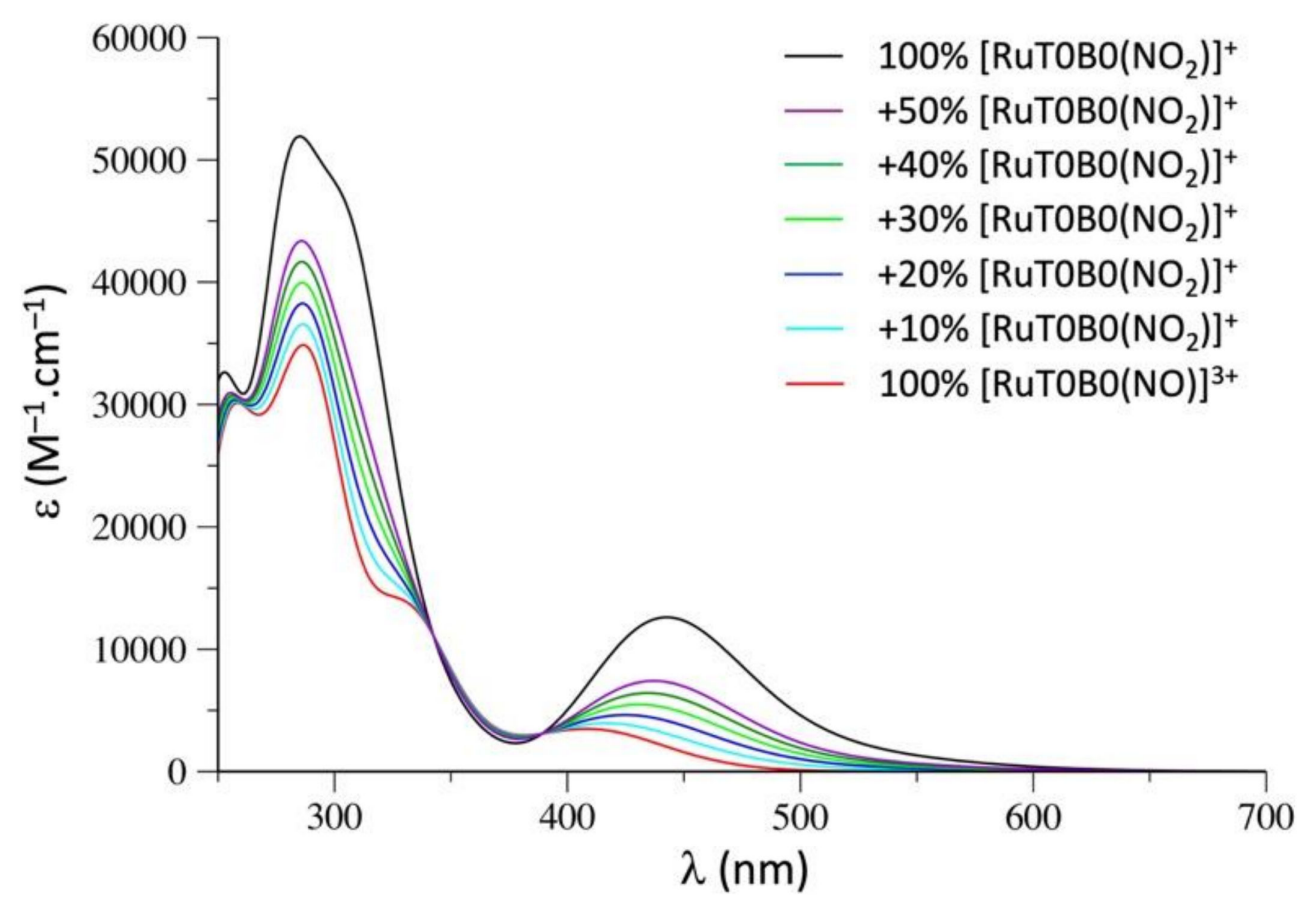
Simulated UV-vis absorption spectra of [RuT0B0(NO)]^3+^ (red curve) and of [RuT0B0(NO_2_)]^+^ (black curve). Mixed spectra adding 10%, 20%, 30%, 40% and 50% of [RuT0B0(NO_2_)]^+^ to [RuT0B0(NO)]^3+^ are shown.

To further validate the assignment of B as the [Ru(NO_2_)] complex, we have computed the UV-vis absorption spectra of [RuT0B0(NO)]^3+^ and of [RuT0B0(NO_2_)]^+^, which appear significantly different according to the experimental data available (Figure 3a). The spectra are shown in Figure 4. The spectrum of [RuT0B0(NO)]^3+^ is characterized by a weak absorption around *λ* = 410 nm and the most intense peak occurs at *λ* = 290 nm with a shoulder around 330 nm. Regarding the [RuT0B0(NO_2_)]^+^ spectrum, there is also an absorption band in the 440 nm region but it is more intense than that of [RuT0B0(NO)]^3+^ by a factor ~3.5. The most intense band peaks at *λ* = 290 nm as for [RuT0B0(NO)]^3+^ but it is broader and more intense. Figure 4 also shows some mixed absorption spectra by combining the [RuT0B0(NO)]^3+^ and [RuT0B0(NO_2_)]^+^ spectra. These results are consistent with the time-evolution of the UV-vis absorption spectra shown in Figure 3a, in which a clear absorption increase is observed for the first and third bands, and a slight decrease of the absorption occurs in the intermediate region, resulting in two isosbestic points.

#### 2.1.3. Origin of Form C

A, B, and C being identified precisely, the relevance of the Scheme 1 becomes questionable. In other words, the origin of C, either from A or from B, must be reconsidered. To clarify this issue, a more careful examination of Figure 2a is necessary. If C arises from the hypothetical form B (initial expectation), the kinetics of its formation must reflect the concentration of B, and the slope of [C] drawn against time (T) (Figure 2b) must be equal to 0 when T = 0. Additional experiments carried out in the very first few minutes of irradiations (see Supporting Information) reveals that the maximum of the slope is precisely observed at T = 0, where the concentration of A is maximum and that of B is equal to 0. Therefore, it becomes clear that C is mainly produced by A instead of B. Nevertheless, and to verify if B takes part in the generation of C, some pure [RuT1B0(NO_2_)](PF_6_) (form B) was prepared (see Experimental Section). An NMR investigation was carried out in the presence of trimethylamine to avoid the formation of A (back reaction in the acid/base equation 3). After two days of irradiation at *λ* = 365 nm, only 10.5% of B was found to be converted to C, however without NO· generation and without interest for biological purpose. Therefore, although the B → C transformation cannot be excluded, one must conclude that C is largely generated by the photoreaction of A.

#### 2.1.4. Origin of Form B

The unexpected production of B from A, observed even in the dark, rises the issue of the mechanism of this reaction. The [Ru(NO)] → [Ru(NO_2_)] reaction depicted in Equation (3) corresponds to the acid/base equilibrium frequently observed in water medium, but we have never observed it in dry solvents, so far. It suggests either an ultra-fast reactivity with traces of moisture in DMSO, or a chemical reactivity with DMSO. A previous investigation has related the stability of [Ru(NO)] species in the presence of water to their ν_NO_ IR frequencies [31]. It was predicted that ν_NO_ frequencies above the 1900–1920 cm^−1^ range implies high degree of positive charge on the nitrosyl, which becomes very sensitive to nucleophilic attack of the solvent, thus leading to the NO → NO_2_ conversion illustrated in Equation (2). We observed here *ν*_NO_ values of 1940 cm^−1^ and 1945 cm^−1^ for [RuT0B0(NO)](PF_6_)_3_ and [RuT1B0(NO)](PF_6_)_3_, respectively, which is consistent with some [Ru(NO_2_)] generation. On the other hand, we have performed our experiments in deuterated DMSO, where the amount of residual water is estimated to be ≤ 0.1%. Under such conditions, the [Ru(NO)] ⇌ [Ru(NO_2_)] reaction cannot be strictly avoided, but it is not observed in other deuterated solvents (e.g., acetonitrile) containing the same amount of residual water, which seems to prove that the nature of the solvent is essential. At this point, it has to be considered that DMSO itself may be an alternative source of oxygen, according to the following equation [32,33,34,35,36]:

(3) $${\left[\mathrm{RuT}1B0(\mathrm{NO})\right]}^{3+}+\mathrm{DMSO}\;⇌\;{\left[\mathrm{RuT}1B0(NO_{2})\right]}^{+}+{\mathrm{Me}}_{2}S$$

However, this possibility which corresponds to a reduction of DMSO is not fully satisfactory in the present case, the [Ru(NO)]^3+^ → [Ru(NO_2_)]^+^ reaction being a non-redox process. On the other hand, DMSO is also known as a Lewis bases which, as potential proton trap, could favor the appearance of OH^−^ and therefore the generation of [Ru(NO_2_)] species, according to the acid-base Equation (2). To verify this hypothesis, we have observed that, in the reference [RuT0B0(NO)]^3+^ complex, where forms A and B can be present in DMSO solution with a very small amount of C, the addition of few drops of HPF_6_, leads to the B → A back conversion. This evolution allows to conclude that the A ⇌ B equilibrium observed in the present species is indeed an acid-base process.

**Scheme 2:**
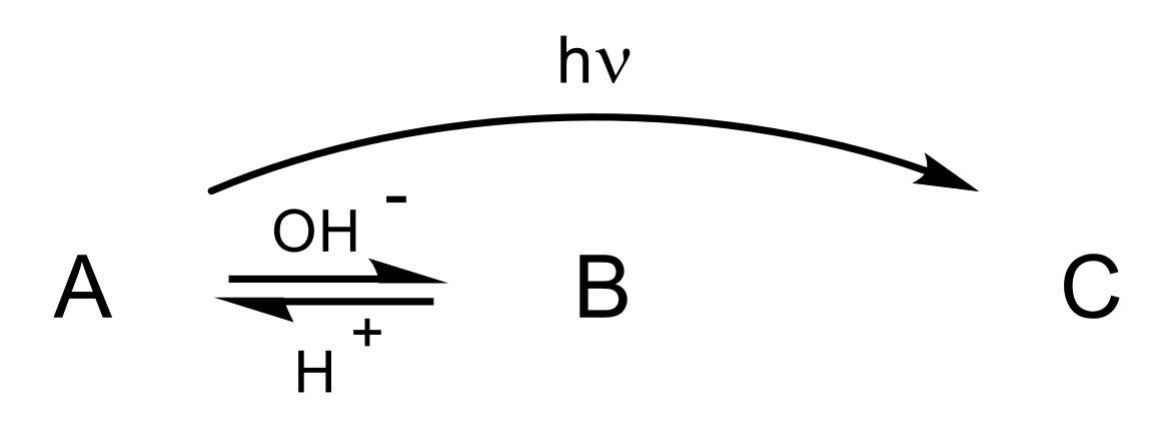
Possible interconversion between A, B, and C, under irradiation of A in DMSO.

In summary, the various investigations undertaken to rationalize the possible conversions between compounds A, B, and C, suggest that the initial A → B → C reaction must be replaced by the following Scheme 2:

### 2.2. Crystal Structures Description

X-ray crystal structures were obtained for [RuT0B0(NO)](PF_6_)_3_ (form A) and [RuT1B0(DMSO_(S)_)](PF_6_)_2_ (form C). The main crystal data are gathered in Table 3 for both compounds.

**Figure 5:**
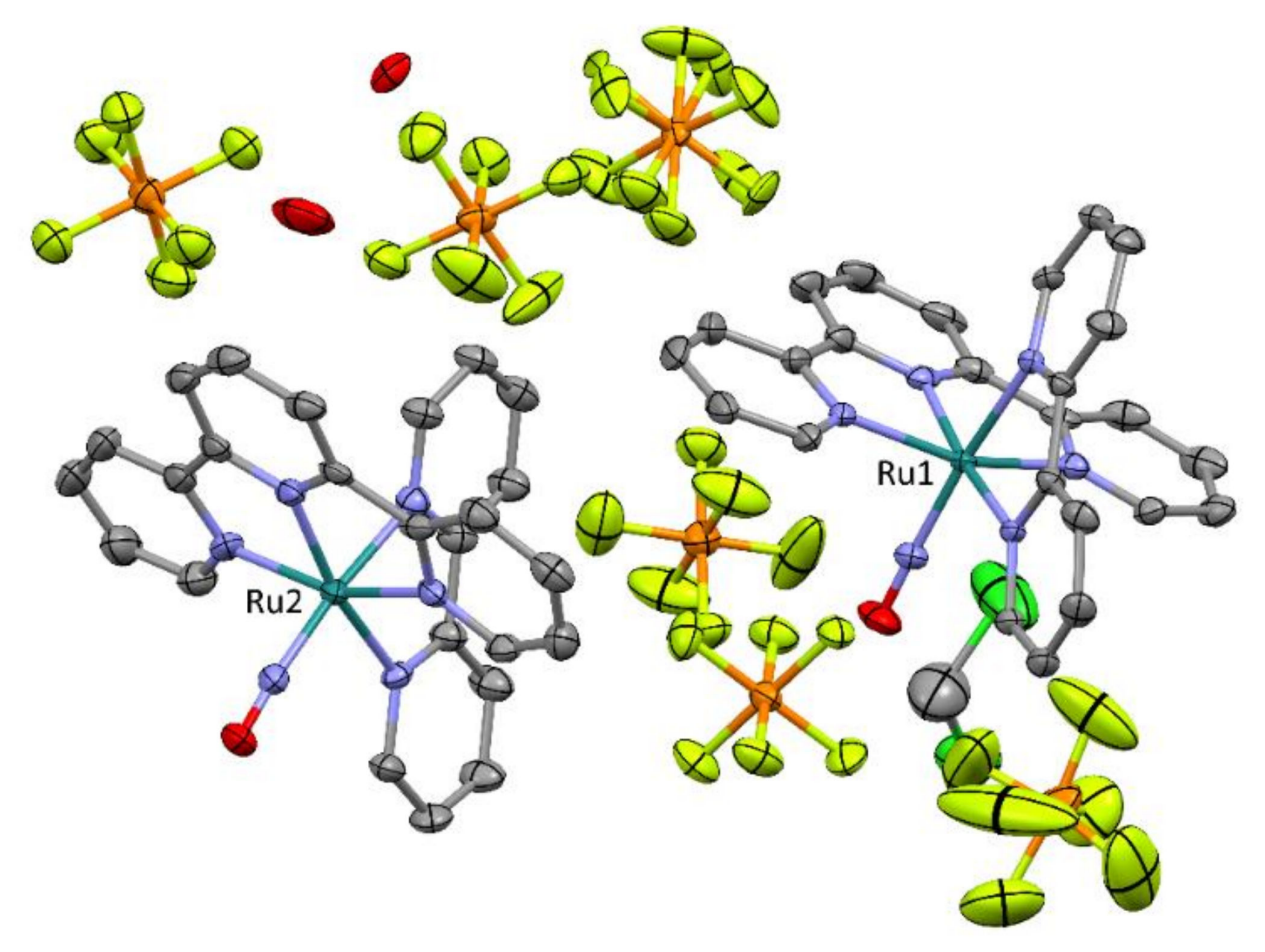
Asymmetric unit for [RuT0B0(NO)](PF_6_)_3_ ∙ ½ CH_2_Cl_2_–H_2_O. Displacement ellipsoids are drawn at the 50% probability level. Hydrogen atoms are omitted for clarity.

[RuT0B0(NO)](PF_6_)_3_ crystallizes in the P2_1_/n monoclinic space group. The asymmetric unit cell, shown in Figure 5, is built up from two ruthenium centers, six PF_6_^−^ counter ions, one molecule of dichloromethane and two molecules of water, the presence of which can be explained by the fact that the solvent used for the diffusion experiment was standard acetonitrile in which some amount of water is always present. The presence of three PF_6_^−^ per ruthenium confirms the 3+ charge of the complex, which corresponds to {Ru-NO}^6^ in the Enemark notation, where 6 is the sum of the electrons present in the *d* orbitals of Ru and those in the π* orbitals of NO [37]. The torsion angle between the mean plane of terpyridine ligand (15 C + 3 N atoms) and that of the bipyridine (10 C + 2 N atoms) is equal to 87.97 deg. and 87.07 deg., for complexes 1 (Ru1) and 2 (Ru2), respectively (Figure 4). The four terpyridine and bipyridine ligands are roughly planar with largest distance to mean plane observed at 0.190 Å, for the C2 atom of the bipyridine of complex 1 (Ru1). Selected bonds and angles for the first coordination sphere of the ruthenium complexes are gathered in Table 4. Although chemically different, the two ruthenium complexes exhibit similar coordination spheres, the shortest Ru-pyridine distance being observed at the central pyridine of the terpyridine ligands. The Ru-N-O angles are close to 180° in both complexes, which indicates for {Ru-NO}^6^ an electronic structure which corresponds to Ru^II^-(NO)^+^ [38].

**Figure 6:**
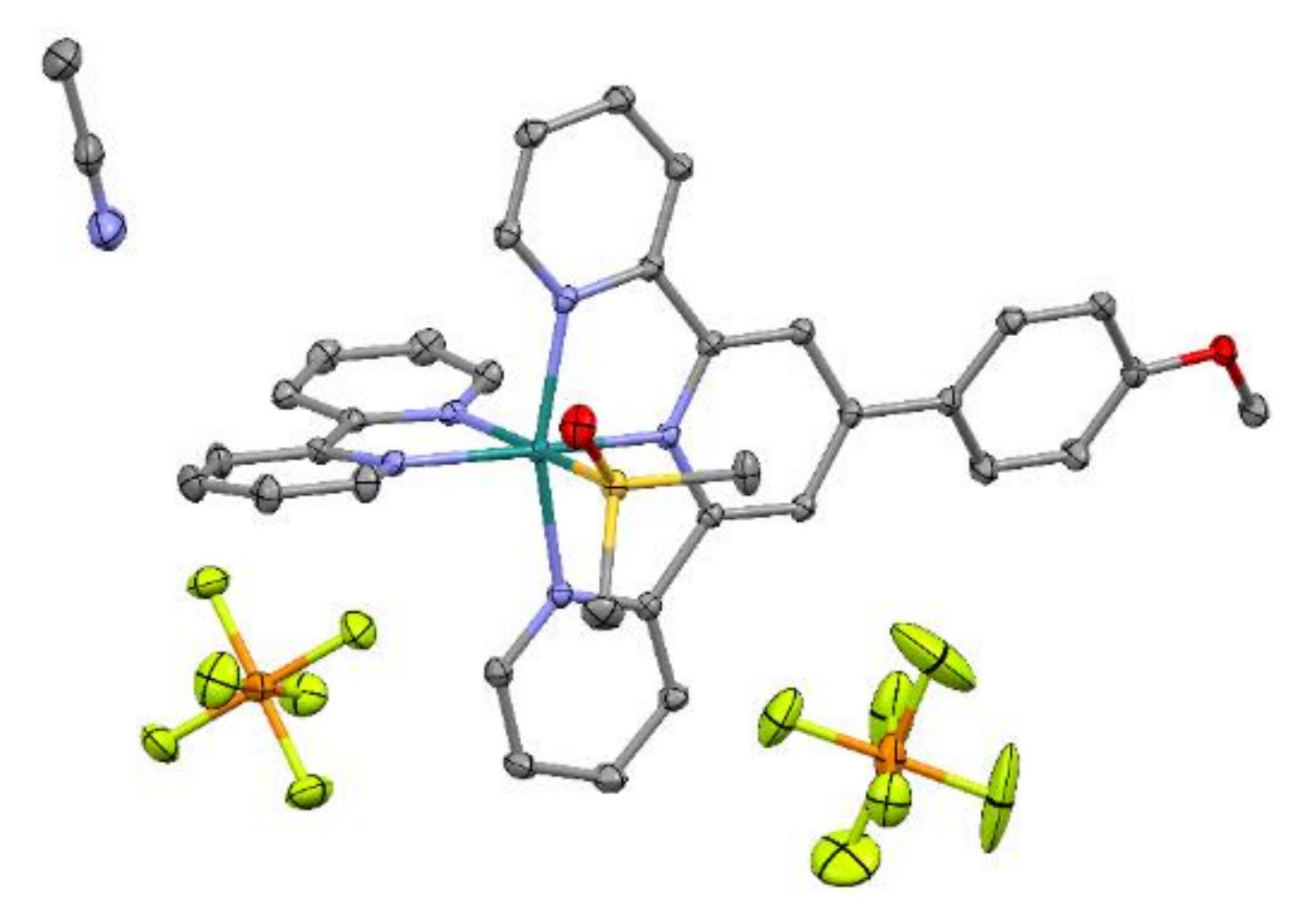
Asymmetric unit for [RuT1B0(DMSO_(S)_)](PF_6_)_2_ ∙ CH_3_CN. Displacement ellipsoids are drawn at the 50% probability level. Hydrogen atoms are omitted for clarity.

[RuT1B0(DMSO_(S)_)](PF_6_)_2_ (form C) crystallizes in the P-1 triclinic space group. The asymmetric unit cell (Figure 6) is built up from one ruthenium complex, two PF_6_^−^, and one molecule of acetonitrile. The presence of two PF_6_^−^ indicate that the oxidation of the metal center corresponds to Ru^II^. The torsion angle between the mean plane of terpyridine ligand and that of the bipyridine is equal to 89.07 deg. As observed in [RuT0B0(NO)]^3+^, the terpyridine and the bipyridine ligands are roughly planar with largest distance to mean plan observed at 0.122 Å, for the C24 atom of the bipyridine. The coordination sphere of [RuT1B0(DMSO_(S)_)]^2+^ is described in Table 4. The S-O bond length of the DMSO ligand is equal to 1.4826(12) Å which is longer than that of 1.46(3) Å observed in the previously reported structure of [RuT0B0(DMSO_(S)_)](SO_3_CF_3_)_2_ [39]. The torsion angle between the methoxyphenyl substituent and the terpyridine is equal to 20.95°, which allows a significant *π* overlap between the rings.

At first, the presence of a Ru^II^ metal center in the photoproduct is surprising, based on Equation (1), which, in the present case, should be written as follows:

(4) $${\left[Ru^{II}\left(NO^{+}\right)\right]}^{3+}+DMSO\;\overset{h\nu }{\to }\;{\left[Ru^{III}\left(DMSO_{\left(S\right)}\right)\right]}^{3+}+NO·$$

An alternative would be to consider the following undesirable equation:

(5) $${\left[Ru^{II}\left(NO^{+}\right)\right]}^{3+}+DMSO\;\overset{h\nu }{\to }\;{\left[Ru^{II}\left(DMSO_{\left(S\right)}\right)\right]}^{2+}+NO^{+}$$

Nevertheless, the observation of NO^+^ release from [Ru(R-tpy)(bpy)(NO)]^3+^ complexes was never observed [23,24,40,41], leading to the conclusion that a fast Ru^III^ → Ru^II^ reduction occurs in the [Ru^III^(solvent)]^3+^ species when five pyridines (tpy + bpy) are present in the coordination sphere, a possibility which has been discussed elsewhere [23].

### 2.3. Photochemical Behavior of [Ru(R-tpy)(bpy)(NO)]^3+^ in DMSO: the Second Step

**Figure 7:**
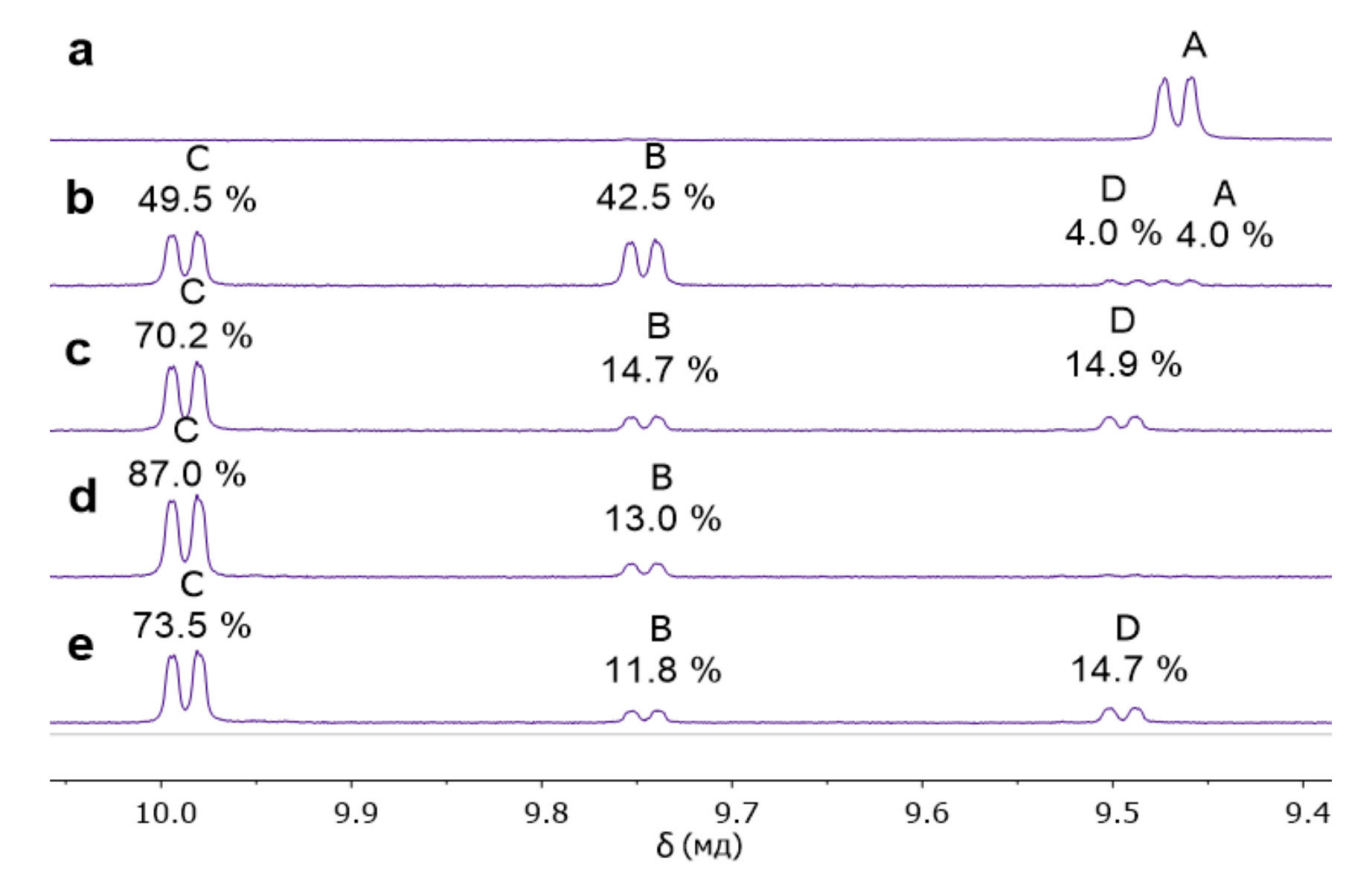
^1^H-NMR spectra in DMSO for [RuT0B0(NO)](PF_6_)_3_ with relative intensities: (**a**) starting compound (form A); (**b**) after 1.5 h of irradiation; (**c**) after 5 h of irradiation; (**d**) after keeping the resulting sample 3 h in the dark; (**e**) after an additional 13 h of irradiation. H-6 is selected as being the most sensitive to the nature of the X ligand present in [Ru(tpy)(bpy)(X)]^n+^ entities.

**Scheme 3:**
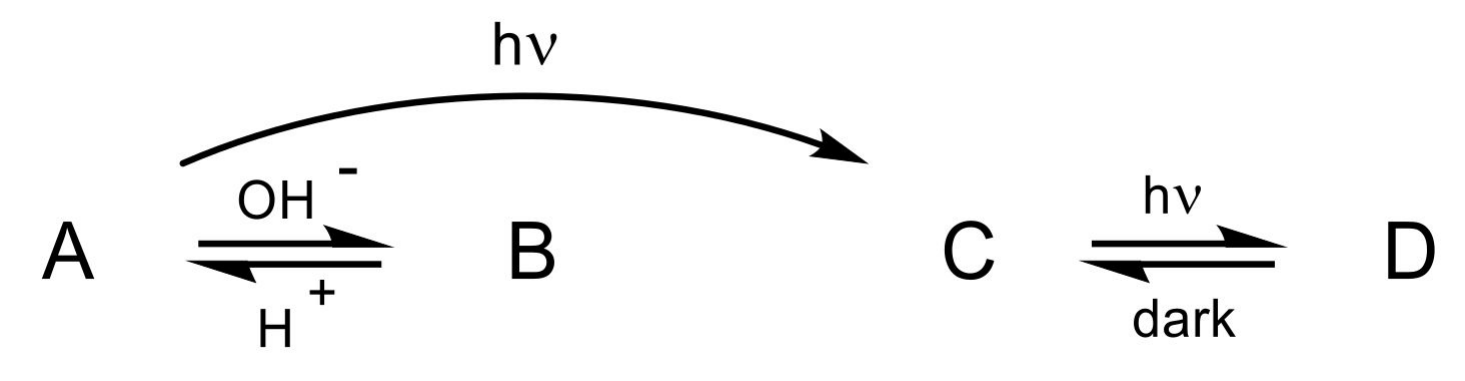
Complete interconversion between forms A, B, C, and D in DMSO.

While the A to C transformation is completed within a couple of hours (Figure 2), it is interesting to observe that an additional but very slow phenomenon takes place under continuous irradiation of the reaction mixture. This general evolution is exemplified in Figure 7, where the ^1^H-NMR signal of atom H-6 of the bipyridine ligand, is presented at various times during an irradiation of a sample of [RuT0B0(NO)](PF_6_)_3_. As the starting [RuT0B0(NO)](PF_6_)_3_ exhibits a single doublet at *δ* = 9.47 ppm (Figure 7a), 1.5 h of irradiation leads to its almost complete conversion to B and C (Figure 7b), an evaluation fully consistent with the experiment described in Figure 2 for [RuT1B0(NO)](PF_6_)_3_. Interestingly, a new doublet identified as form D in this figure appears at 9.49 ppm. However, its concentration remains very low (4%). After 5 h of irradiation (Figure 7c), most of the conversion of A to C is achieved, the B → C reaction being very slow. Nevertheless, the concentration of the new form D rises to 14.9%. At this point, when the reaction mixture is kept in the dark for 3 h (Figure 7d), the new form D completely disappears, which indicate a D → C transformation in the dark. Interestingly, an additional irradiation of 13 h leads to the restoration of the situation observed in Figure 7c. Altogether, these experiments indicate that D is formed by irradiation of C, the process being slow and completely reversible in the dark. The overall photoreaction process is summarized in Scheme 3.

It can be inferred from the above experiment that D is a photo-isomerized form of [RuT0B0(DMSO_(S)_)]^2+^, which can be identified as [RuT0B0(DMSO_(O)_)]^2+^. Indeed, the Ru-S to Ru-O linkage isomerization of ruthenium-polypyridine sulfoxide complexes has previously been reported, and thoroughly reviewed [20,42,43]. The assignment of the D form is here supported by our theoretical calculations of the ^1^H-NMR chemical shifts. The decrease of the chemical shift (shielding) for H-6 on going from C to D is computed to be 0.6 ppm for the T1B0 complex (Table 2), which is in good agreement with the shielding of ca. 0.5 ppm observed experimentally in the T0B0 complex (Figure 7).

A literature survey reveals that the S → O linkage isomerization of Ru-DMSO species was first triggered electrochemically [44,45]. The observed tendency is that polarizable Ru^II^ metal centers exhibit a preference for the *π*-acceptor S-bonded DMSO, while O-bonded forms are stabilized by Ru^III^ species. In the case of the present investigation and as the metal centers are in oxidation state II, the possibility for linkage isomerization to the metastable Ru-DMSO_(O)_ isomers is naturally addressed. In the case of [RuT0B0(DMSO_(S)_)]^2+^, the complete characterization of D is not possible because this species appears rather unstable and is always present as a small amount in the reaction mixture.

In an attempt to try to find a correlation between the rate of Ru-DMSO_(S)_ → Ru-DMSO_(O)_ photo-isomerization and the electronic structures of the complexes, [RuT0B0(DMSO_(S)_)]^2+^ and [RuT1B0(DMSO_(S)_)]^2+^ were investigated together with additional ruthenium complexes. In these new species, so called [RuT2B0(DMSO_(S)_)]^2+^ and [RuT3B0(DMSO_(S)_)]^2+^ (Figure 1), Br and NO_2_ substituents are present, respectively, to modulate the intramolecular charge transfer capabilities. These investigations were carried out on pure complexes in the C form (Ru-DMSO_(S)_), obtained by direct chemical synthesis (see Materials and Methods). The irradiations were performed for several hours, in order to reach the Ru-DMSO_(S)_) ⇌ Ru-DMSO_(O)_ equilibrium, which was observed to be achieved within one hour for the four complexes under investigation. The Ru-DMSO_(O)_/Ru-DMSO_(S)_ ratios are presented in Table 5. It appears from these experiments that the more donating substituent (MeO in [RuT1B0(DMSO_(S)_)]^2+^) leads to the lowest ratio of isomerization (25%), while the more withdrawing substituent (NO_2_ in [RuT3B0(DMSO_(S)_)]^2+^) leads to the highest ratio (46%). This result is counterintuitive with respect to the natural assumption that stronger charge transfers toward the Ru-DMSO fragment should encourage more efficient linkage isomerizations in these species. However, it is known that triplet metal centered d-d states play a crucial role in the photo-isomerization mechanism of ruthenium sulfoxide complexes [15,18,46]. In addition, the respective absorption properties of the Ru-DMSO_(O)_ and Ru-DMSO_(S)_ will play a role in the photoconversion yield. Thus, this simple reasoning cannot be used.

In an alternative approach the DMSO_(O)_/DMSO_(S)_ ratio can be tentatively related to the absorbance of both species at the wavelength of irradiation (*λ* = 365 nm). Along this line, and assuming that the quantum yield of photo-isomerization is similar for DMSO_(S)_ $\overset{h\nu }{\to }$ DMSO_(O)_ and for DMSO_(O)_ $\overset{h\nu }{\to }$ DMSO_(S)_ photoreactions, the equilibrium is expected when the absorbance (extinction coefficient (*ε*) × concentration) is the same for both DMSO_(O)_ and DMSO_(S)_ species, leading to the following equation:

*ε*(Ru-DMSO_(S)_) × [Ru-DMSO_(S)_] ≈ *ε*(Ru-DMSO_(O)_) × [Ru-DMSO_(O)_] (6)

**Figure 8:**
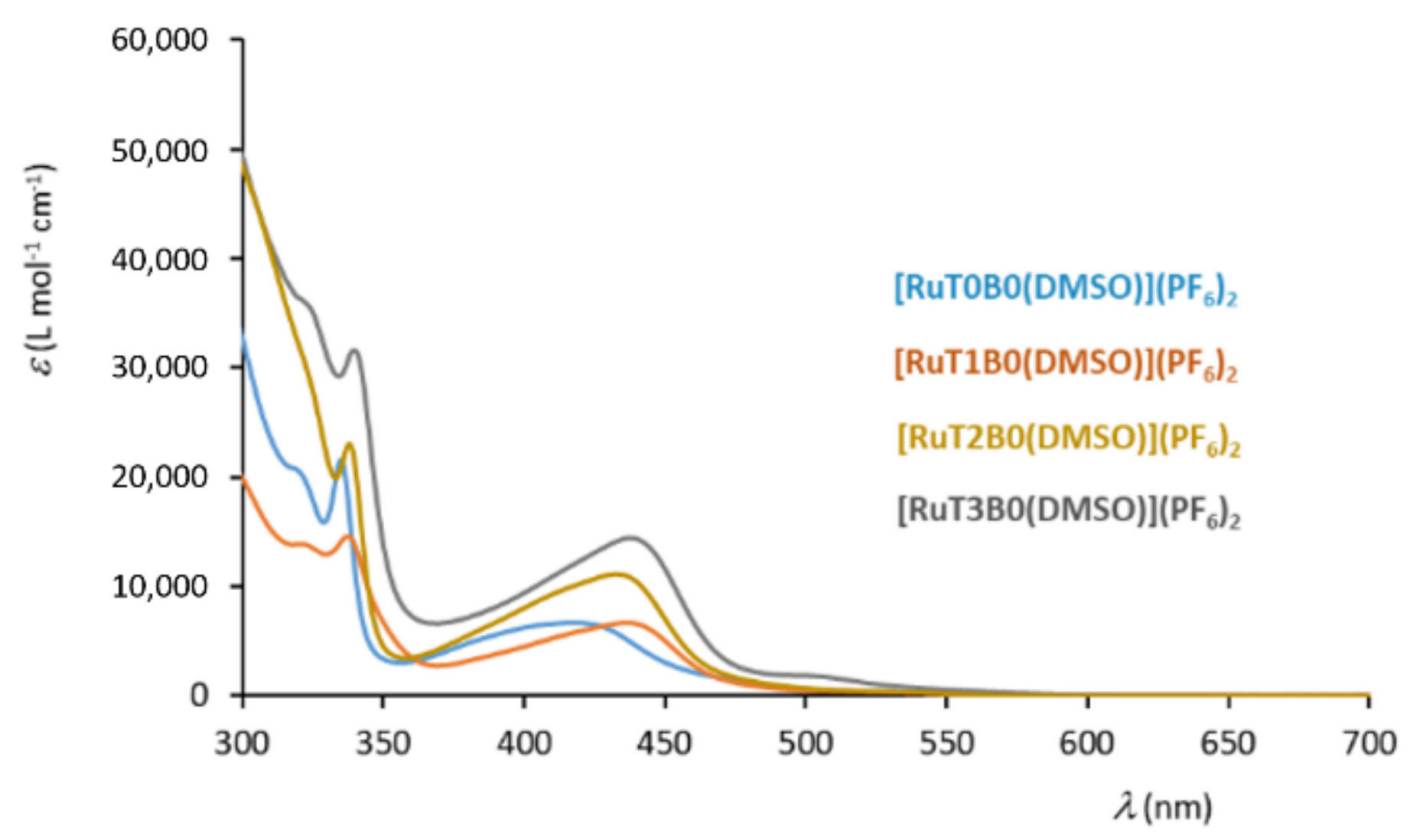
UV-visible spectra in acetonitrile for the four Ru-DMSO_(S)_ complexes.

To verify this hypothesis, the UV-visible spectra of Ru-DMSO_(S)_, and Ru-DMSO_(O)_ complexes have to be examined. No UV-visible data being available for the Ru-DMSO_(O)_ isomers, which cannot be isolated, the comparison had to be carried out on computed spectra. Nevertheless, the accuracy of the computation can be tested with the experimental spectra available for the Ru-DMSO_(S)_ forms, gathered in Figure 8.

Experimentally, the four Ru-DMSO_(S)_ compounds exhibit a low energy transition with absorption maxima in the *λ* = 415–440 nm range, more intense transitions being present at higher energy. This general feature is confirmed by the simulated spectra (Supplementary Materials), for which a slight blue shift not exceeding 1500 cm^–1^ (<0.2 eV) is obtained for the low-lying transitions compared to the experimental spectra. Therefore, the agreement between computations and experiments appears satisfactory, which supports the reliability of the computational approach. The main features of the computed spectra are shown in Table 6.

The computed extinction coefficients at 365 nm lead to the Ru-DMSO_(O)_/Ru-DMSO_(S)_ ratio gathered in Table 5. The values substantially agree with the experiment and confirm that the most electron withdrawing substituent (NO_2_-phenyl) leads to the more efficient Ru-DMSO_(S)_ → Ru-DMSO_(O)_ isomerization process. With this respect, the difference in absorption occurring during the linkage isomerization appears to be a key parameter to explain the concentration of Ru-DMSO_(O)_ at the equilibrium.

## 3. Materials and Methods

### 3.1. Starting Materials and Equipment

The starting chemicals 2-acétylpyridine, NH_4_PF_6_ and 2,2′:6′,2″-terpyridine were purchased from Alfa-Aesar (Haverhill, MA, USA). Hexafluorophosphoric acid (~65% in H_2_O) was from Sigma-Aldrich (Tharabie, France), 2,2′-bipyridine from TCI (TCI Europe N.V., Zwijndrecht, Belgium), RuCl_3_xH_2_O from Strem Chemicals (Bischheim, France), 4-methoxybenzaldehyde, triethylamine and trimethylamine from Acros Organics (Fisher Scientific, Geel, Belgium), and NaNO_2_ from Fluka (Saint-Quentin-Fallavier, France). Chemicals and solvents were analytical grade and used without further purification. [RuT0B0(NO)](PF_6_)_3_, and [RuT1B0(NO)](PF_6_)_3_ (forms A) were prepared following the procedure previously reported by Meyer [47,48], and by our group [23], respectively. [RuT1B0(NO_2_)](PF_6_) (form B) was prepared chemically as an intermediate towards the synthesis of [RuT1B0(NO)](PF_6_)_3_ [23]. Bromophenylterpyridine [49] and nitrophenylterppyridine [50] were prepared as previously described.

Elemental analyses were performed at LCC with a 2400 series II Instrument (Perkin–Elmer, Waltham, MA, USA). UV-Vis spectra were obtained on a Perkin Elmer Lambda 35 UV-Vis spectrometer (Perkin–Elmer, Waltham, MA, USA). Irradiation experiments were achieved on 8–9 milligrams on ruthenium complexes dissolved in 0.6 mL of DMSO-*d_6_* in a NMR tube with a LED-lamp operating at *λ* = 365 nm or 395 nm, under constant fanning. The experiments were carried out in the NMR room, and the composition of the reaction mixtures were recorded periodically by ^1^H-NMR spectroscopy. The time required for transferring the samples to the spectrometer were restricted to few seconds. The ^1^H-NMR spectra were recorded in DMSO-*d_6_*, on an Avance 400 Bruker spectrometer (Bruker-France, Wisembourg 67160). The complete assignment for the ruthenium complexes are provided in Supplementary Materials (Figures S1,S2). In the case of [RuT1B0(NO)](PF_6_)_3_, the completeness of the A → C transformation (95%) was achieved after a time of irradiation of about 7 h. The solution was then transferred into a round-bottomed flask, and evaporated to dryness under vacuum at the Schlenk line, with moderate heating (40–50 °C). The resulting solid was dissolved in 2 mL of acetonitrile, filtered, transferred into a vial and placed in a large beaker in the presence of a flask, filled with a large amount of diethyl ether. Single crystals suitable for X-ray analyses appear within a few days after slow diffusion of diethyl ether into the concentrated solution of C. For the investigation of the direct C → D transformations (Table 5), the pure ruthenium-DMSO_(S)_ complexes (form C) were diluted to 3 × 10^−3^ mol·L^−1^ and fully irradiated for several hours in a vial under stirring, and checked regularly by ^1^H-NMR. The C/D equilibrium was reached within one hour of irradiation, in any cases.

### 3.2. Synthesis of Ru-DMSO Complexes (Form C)

The four compounds were synthesized from the [Ru(R-tpy)bpyCl](Cl) precursors: [RuT0B0(Cl)](Cl) [51] and [RuT1B0(Cl)](Cl) [2] were previously reported in the literature. [RuT2B0(Cl)](Cl) and [RuT3B0(Cl)](Cl) were synthesized according to the methodology previously reported for [RuT1B0(Cl)](Cl) [23].

[RuT2B0Cl](Cl). Starting materials: [RuT2Cl_3_] (425 mg, 0.69 mmol), 2,2′-bipyridine (108 mg, 0.69 mmol), LiCl (165 mg, 3.9 mmol), trimethylamine (0.82 mL, 5.9 mmol), EtOH (48 mL), H_2_O (16 mL). Yield: 390 mg, 68%. ^1^H-NMR (400 MHz, DMSO-*d_6_*) δ 10.11 (dd, *J* = 5.7, 1.5 Hz, 1H), 9.22 (s, 2H), 8.95 (dd, *J* = 8.3, 5.0 Hz, 3H), 8.66 (d, *J* = 8.1 Hz, 1H), 8.41–8.28 (m, 3H), 8.14–7.97 (m, 5H), 7.79 (td, *J* = 7.8, 1.4 Hz, 1H), 7.64 (dd, *J* = 5.6, 1.5 Hz, 2H), 7.41 (ddd, *J* = 7.4, 5.6, 1.3 Hz, 3H), 7.08 (ddd, *J* = 7.3, 5.7, 1.3 Hz, 1H).

[RuT3B0Cl](Cl). Starting materials: [RuT3Cl_3_] (626 mg, 1.1 mmol), 2,2′-bipyridine (174 mg, 1.1 mmol), LiCl (351 mg, 8.4 mmol), trimethylamine (1.31 mL, 9.4 mmol), EtOH (90 mL), H_2_O (30 mL). Yield: 578 mg, 66%. ^1^H-NMR (400 MHz, methanol-*d*_4_) δ 10.26 (ddd, *J* = 5.6, 1.6, 0.8 Hz, 1H), 9.04 (s, 2H), 8.84–8.78 (m, 1H), 8.75–8.70 (m, 2H), 8.54–8.49 (m, 3H), 8.45–8.39 (m, 2H), 8.39–8.32 (m, 1H), 8.06 (ddd, *J* = 7.6, 5.6, 1.3 Hz, 1H), 8.00–7.93 (m, 2H), 7.80–7.72 (m, 3H), 7.47–7.42 (m, 1H), 7.38 (ddd, *J* = 7.6, 5.5, 1.3 Hz, 2H), 7.06 (ddd, *J* = 7.3, 5.8, 1.4 Hz, 1H).

#### 3.2.1. [RuT0B0(DMSO)_(S)_](PF_6_)_2_

[RuT0B0(Cl)](Cl) was first transformed to [RuT0B0(Cl)](PF_6_) by metathesis, using a 20-fold excess of NH_4_PF_6_ in water. Then, and following the methodology described by Root and Deutsch [45], [RuT0B0(Cl)](PF_6_) (83 mg, 0.12 mmol) and AgPF_6_ (62 mg, 0.24 mmol), were added to a two-necked flask and connected to a Schlenk line. The flask was filled with argon and deaerated DMSO (8 mL) was added. The resulting solution was stirred for 45 h at 80 °C, then cooled down to room temperature. The AgCl precipitate formed during the reaction was filtered off. Finally, DMSO was evaporated under vacuum resulting of a light-orange powder, which was thoroughly rinsed by toluene, to remove the unreacted AgPF_6_. Yield: 77 mg (0.09 mmol); φ = 75%. ^1^H-NMR (400 MHz, DMSO-*d*_6_) δ 10.03–9.96 (m, 1H), 8.95 (dd, *J* = 8.3, 5.1 Hz, 3H), 8.81–8.74 (m, 3H), 8.59 (t, *J* = 8.1 Hz, 1H), 8.46 (td, *J* = 7.7, 1.3 Hz, 1H), 8.20 (td, *J* = 7.9, 1.5 Hz, 2H), 8.15–8.02 (m, 2H), 7.85–7.78 (m, 2H), 7.53 (ddd, *J* = 7.0, 5.6, 1.3 Hz, 2H), 7.39–7.30 (m, 1H), 7.16–7.09 (m, 1H). IR: ν (S=O) =1103 cm^−1^.

#### 3.2.2. [RuT1B0(DMSO)_(S)_](PF_6_)_2_

The synthesis was achieved following the procedure described above for [RuT0B0(DMSO)_(S)_](PF_6_)_2_. Starting materials: [RuT1B0(Cl)](PF_6_) (200 mg 0.25 mmol), AgPF_6_ (126 mg, 0.5 mmol), DMSO (8 mL).Yield: 204 mg; 85%. ^1^H-NMR (400 MHz, DMSO-*d*_6_) δ 10.06–9.99 (m, 1H), 9.28 (s, 2H), 9.04 (dd, *J* = 8.1, 1.2 Hz, 2H), 8.94 (d, *J* = 8.1 Hz, 1H), 8.77 (d, *J* = 8.2 Hz, 1H), 8.45 (td, *J* = 7.9, 1.5 Hz, 1H), 8.41–8.35 (m, 2H), 8.22 (td, *J* = 7.9, 1.5 Hz, 2H), 8.14–8.02 (m, 2H), 7.86–7.78 (m, 2H), 7.53 (ddd, *J* = 7.1, 5.6, 1.3 Hz, 2H), 7.35 (ddd, *J* = 7.3, 5.7, 1.2 Hz, 1H), 7.32 – 7.23 (m, 3H). IR: ν (S=O) =1101 cm^−1^. MS: *m*/*z*=337.33 (*m*/*z*_theor_ ([Ru(MeOPhtpy)bpyDMSO]^2+^) = 337.56); 819.42 (m/z_theor_ ([Ru(MeOPhtpy)bpyDMSO](PF_6_)^+^) = 820.08). Anal. Calcd. for C_34_H_31_F_12_N_5_O_2_P_2_RuS: C, 42.33; H, 3.24; N, 7.26. Found: C, 42.63; H, 3.33; N, 6.91.

#### 3.2.3. [RuT2B0(DMSO)_(S)_](PF_6_)_2_

The synthesis was achieved following the procedure described above for [RuT1B0(DMSO)_(S)_](PF_6_)_2_. Starting materials: [Ru(BrPhtpy)bpyCl]PF_6_ (283 mg, 0.34 mmol), AgPF_6_ (174 mg, 0.68 mmol), DMSO (30 mL). Yield: 311 mg (0.31 mmol) 92%. ^1^H-NMR (400 MHz, DMSO-*d*_6_) δ 10.08–10.00 (m, 1H), 9.35 (s, 2H), 9.05 (d, *J* = 8.1 Hz, 2H), 8.95 (d, *J* = 8.1 Hz, 1H), 8.78 (d, *J* = 8.4 Hz, 1H), 8.52–8.42 (m, 1H), 8.40–8.32 (m, 2H), 8.25 (td, *J* = 7.9, 1.5 Hz, 2H), 8.16–8.03 (m, 2H), 8.03–7.95 (m, 2H), 7.84 (dt, *J* = 5.5, 1.3 Hz, 2H), 7.56 (ddd, *J* = 7.0, 5.6, 1.3 Hz, 2H), 7.35 (ddd, *J* = 7.3, 5.7, 1.3 Hz, 1H), 7.31–7.24 (m, 1H). IR: ν (S=O) =1092 cm^−1^ MS: *m*/*z* = 362.25 (*m*/*z*_theor_([Ru(BrPhtpy)bpyDMSO]^2+^) = 361.51); 869.33 (m/z_theor_ ([Ru(BrPhtpy)bpyDMSO](PF_6_)^+^) = 868.98). Anal. Calcd. for C_33_H_28_BrF_12_N_5_O_2_P_2_RuS: C, 39.10; H, 2.78; N, 6.91. Found: C, 39.13; H, 3.12; N, 6.53.

#### 3.2.4. [RuT3B0(DMSO)_(S)_](PF_6_)_2_

The synthesis was achieved following the procedure described above for [RuT0B0(DMSO)_(S)_](PF_6_)_2_. Starting materials: [Ru(NO_2_Phtpy)bpyCl]PF_6_ (101 mg, 0.13 mmol), AgPF_6_ (66 mg, 0.26 mmol), DMSO (10 mL). Yield: 109 mg (0.11 mmol 87%). ^1^H-NMR (400 MHz, DMSO-*d*_6_) δ 10.04 (dd, *J* = 5.5, 1.3 Hz, 1H), 9.42 (s, 2H), 9.09–9.03 (m, 2H), 8.95 (d, *J* = 8.0 Hz, 1H), 8.77 (d, *J* = 8.2 Hz, 1H), 8.61 (d, *J* = 0.7 Hz, 4H), 8.46 (td, *J* = 7.9, 1.5 Hz, 1H), 8.26 (td, *J* = 7.9, 1.5 Hz, 2H), 8.16–8.10 (m, 1H), 8.09–8.03 (m, 1H), 7.87–7.81 (m, 2H), 7.57 (ddd, *J* = 7.7, 5.5, 1.3 Hz, 2H), 7.36–7.30 (m, 1H), 7.30–7.25 (m, 1H). IR: ν (S=O) = 1091 cm^−1^. MS: *m*/*z* = 344.83 (*m*/*z*_theor_([Ru(NO_2_Phtpy)bpyDMSO]^2+^) = 345.05). Anal. Calcd. for C_33_H_28_F_12_N_6_O_3_P_2_RuS: C, 40.46; H, 2.88; N, 8.58. Found: C, 40.62; H, 2.68; N, 8.30.

### 3.3. Synthesis of Form D, by Photo-Isomerization of Form C

Although the photo-generated Ru-DMSO_(O)_ complexes were not isolated, their ^1^H-NMR spectra (400 MHz, DMSO-*d_6_*) are accessible from the C/D mixture (see Supplementary Materials). For [RuT0B0(DMSO)(S)](PF_6_)_2_: δ 9.49 (d, *J* = 5.5 Hz, 1H), 8.98–8.89 (m, 3H), 8.80 (d, *J* = 8.2 Hz, 3H), 8.63 (d, *J* = 8.1 Hz, 1H), 8.39 (d, *J* = 8.0 Hz, 2H), 8.11 (m, 2H), 7.76 (m, 1H), 7.72 (dd, *J* = 5.2, 1.4 Hz, 2H), 7.48 (ddd, *J* = 7.4, 5.6, 1.3 Hz, 2H), 7.28 (d, 1H), 7.07 (t, 1H).

For [RuT1B0(DMSO)_(S)_](PF_6_)_2_: δ 9.52 (d, *J* = 5.5 Hz, 1H), 9.29 (s, 2H), 9.06 (d, 2H), 8.95 (m, 1H), 8.63 (d, *J* = 8.2 Hz, 1H), 8.14–8.04 (m, 5H), 7.78 (t, 1H), 7.72 (d, *J* = 5.4 Hz, 2H), 7.50–7.45 (t, 2H), 7.38 (d, 1H), 7.27 (m, 3H), 7.07 (t, 1H).

For [RuT2B0(DMSO)_(S)_](PF_6_)_2_: δ 9.52 (d, *J* = 5.5 Hz, 1H), 9.36 (s, 2H), 9.06 (d, *J* = 7.8 Hz, 2H), 8.97 (d, *J* = 9.5 Hz, 1H), 8.64 (d, *J* = 8.1 Hz, 1H), 8.39 (d, 2H), 8.19–8.14 (m, 3H), 7.96 (d, 2H), 7.79 (t, *J* = 7.8 Hz, 1H), 7.76–7.71 (m, 2H), 7.52–7.47 (m, 2H), 7.37 (m, 2H), 7.10–7.03 (t, 1H).

For [RuT3B0(DMSO)_(S)_](PF_6_)_2_: δ 9.52 (d, *J* = 5.5 Hz, 1H), 9.44 (s, 2H), 9.08 (d, *J* = 9.2 Hz, 2H), 8.98 (d, 1H), 8.69–8.54 (m, 5H), 8.46 (t, *J* = 7.6 Hz, 1H), 8.21–8.10 (m, 3H), 7.82–7.77 (t, 1H), 7.76–7.73 (d, 2H), 7.52 (ddd, *J* = 7.1, 5.5, 1.3 Hz, 2H), 7.36 (d, *J* = 6.1 Hz, 1H), 7.09–7.03 (m, 1H).

### 3.4. X-ray Studies

Single crystals of [RuT0B0(NO)](PF_6_)_3_ (form A) where obtained by slow diffusion of dichloromethane into a concentrated solution of the compound (8–9 mg) in acetonitrile. Crystals of [RuT1B0(DMSO_(S)_)](PF_6_)_3_ (form C) were obtained after extensive irradiation of [RuT1B0(NO)](PF_6_)_3_ (form A, 8 mg in 0.5 mL of DMSO), with a LED lamp at *λ* = 395 nm. After 6 h, the compound was found to be converted to form C at 94%, by ^1^H-NMR. The solvent was removed under vacuum with a Schlenk line. Slow diffusion of diethyl ether into a solution of the resulting solid dissolved in acetonitrile led to the formation of single crystals of form C.

Intensity data were collected at low temperature on an Apex2 Bruker diffractometer (equipped with a 30W air-cooled microfocus Mo source (λ = 0.71073 Å). The structures were solved using SUPERFLIP [52] and refined by means of least-squares procedures using the programs of the PC version of CRYSTALS [53]. Atomic scattering factors were taken from the international tables for X-ray crystallography. All non-hydrogen atoms were refined anisotropically. The H atoms were located in a difference map, but those attached to carbon atoms were repositioned geometrically. The H atoms were refined with riding constraints. Absorption corrections were introduced using the program MULTISCAN [54]. The crystal structures have been deposited with the Cambridge Crystallographic Data Center (CCDC 1992867 for [RuT0B0(NO)](PF_6_)_3_, and CCDC 1992868 for [RuT1B0(DMSO_(S)_)](PF_6_)_3_).

### 3.5. Computational Studies

All the structures were optimized using density functional theory (DFT) with the standard hybrid B3LYP functional [55] complemented by the D3 version of Grimme’s dispersion correction with Becke-Johnson damping [56]. A triple-ζ def2-TZVP basis set was used on all atoms and the corresponding pseudo-potential was used on the Ru center [57]. All the geometry optimizations were carried out within the polarizable continuum model (PCM) as implemented in Gaussian 09 [58] to describe the DMSO solvent. Calculations of vibrational harmonic frequencies were systematically performed in order to characterize the nature of the stationary points (minima or saddle points). UV-vis absorption spectra were simulated at the time-dependent DFT (TD-DFT) level using the same functional and same basis set as described above. The DMSO solvent was also described with PCM. The convoluted spectra were obtained using a Gaussian with a half-width at half-maximum of 1800 cm^–1^ at each vertical transition given by TD-DFT. The chemical shifts were computed within the gauge including atomic orbital (GIAO) method [59,60,61]. These calculations were performed with B3LYP and PCM using an extended quadruple-ζ def2-QZVPP basis set on all atoms and the corresponding pseudo-potential for Ru [57]. Because of the presence of several conformers for some species (for example C was found to have four rotamers), the chemical shifts were computed as a weighted average of the chemical shifts for these various conformers using Boltzmann averaging.

## 4. Conclusions

[Ru(MeOPh-tpy)(bpy)(NO)](PF_6_)_3_ is an intriguing compound which undergoes a multi-step chemical and photochemical behavior in DMSO. By combining various experimental analyses (X-ray crystallography, ^1^H-NMR, UV-vis absorption spectroscopy) and theoretical calculations, we were able to identify all the resulting products of these reactions. First, this complex exhibits an extreme sensitivity to trace of water leading to a reversible acid-base Ru-NO → RuNO_2_ evolution stimulated by the intrinsic basic character of DMSO. Under irradiation, [Ru(MeOPh-tpy)(bpy)(NO)]^3+^ is subjected to a NO· release, with the concomitant generation of [Ru(MeOPh-tpy)(bpy)(DMSO_(S)_)]^2+^ as a photoproduct, in which the DMSO ligand is linked to the ruthenium by the sulfur atom. Under continued irradiation, the resulting Ru-DMSO complex undergoes a further, but reversible, isomerization process which leads to an equilibrium between the Ru-DMSO_(S)_ and Ru-DMSO_(O)_ species. By using different substituents attached to the tpy ligands, we were able to modulate the photoreactivity of this complex and of the photosubstitution product. In particular, by removing the electron-donor MeOPh group, the NO· photorelease is quenched to the profit of RuNO_2_ formation. On the other hand, electron-withdrawing substituents favor the S→O linkage photo-isomerization of the Ru-DMSO_(S)_ photoproduct. Thus, our study brings further evidences of the very rich photochemistry of ruthenium nitrosyl complexes, with the most significant and positive feature that the presence of DMSO does not affect the capability of [Ru(tpy)(bpy)(NO)]^3+^ for the NO release (form A → form C + NO·), taking into account the frequent use of DMSO in practical biological test.

## Figures and Tables

**Table 1:** Chemical shifts of protons in position 6 on the bipyridine ligands, in the case of compounds with unsubstituted ([RuT0B0(X)](PF_6_)_3_) and methoxyphenyl-substituted ([RuT1B0(X)](PF_6_)_3_) terpyridine.

Ligand X	[RuT0B0(X)](PF_6_)_3_	[RuT1B0(X)](PF_6_)_3_
Cl^−^	10.20 ppm	10.26 ppm
NO_2_^−^ (form B)	9.755 ppm	9.77 ppm ^a^
NO (form A)	9.47 ppm	9.50 ppm ^a^

^a^ data from the NMR spectra shown in Figure 2.

**Table 2:** Relative chemical shift of protons in position 6 on the bipyridine ligands with respect to the A form of complex [RuT1B0(NO)]^3+^.

Structures	Δ(δ^H-6^)
[RuT1B0(η^2^-NO)]^3+^	−0.33 ppm
[RuT1B0(ON)]^3+^	−0.14 ppm
[RuT1B0(NO_2_)]^+^	+0.93 ppm
[RuT1B0(DMSO_(S)_)]^2+^	+1.34 ppm
[RuT1B0(DMSO_(O)_)]^2+^	+0.74 ppm

**Table 3:** Crystal data for [RuT0B0(NO)](PF_6_)_3_, and [RuT1B0(DMSO_(S)_)](PF_6_)_2_.

	[RuT0B0(NO)](PF_6_)_3_ ½ CH_2_Cl_2_–H_2_O	[RuT1B0(DMSO_(S)_)](PF_6_)_2_ CH_3_CN
Chemical formula	C_25.5_H_22_ClF_18_N_6_O_2_P_3_Ru	C_36_H_34_F_12_N_6_O_2_P_2_RuS_1_
Molecular weight (g/mol)	1015.90	1005.76
Crystal system	monoclinic	triclinic
Space group	P2_1_/n	P-1
a (Å)	19.2309(9)	12.6477(6)
b (Å)	19.2739(10)	13.2076(5)
c (Å)	19.7628(10)	13.2282(7)
a (deg)	90	97.4744(14)
b (deg)	106.5407(17)	109.0108(14)
g (deg)	90	106.0250(14)
V (Å^3^)	7022.0(6)	1948.27(16)
Z	8	2
T (K)	110	113
μ (MoKα)	0.793	0.639
D_calc_ (g/cm^3^)	1.92	1.71
T (K)	110	113
Reflect. measured	173754	99152
2θ range	1.30° ≤ 2θ ≤ 28.34°	1.65° ≤ 2θ ≤ 30.61°
Reflect. unique	17439	11796
Rint	0.0777	0.0432
Refinement on	F	F
Nb Parameters	1048	541
R1 (I > nσ(I))	0.0767, n = 2.5	0.0294, n = 3
wR2 (I > nσ(I))	0.0964, n = 2.5	0.0305, n = 3
Residual electron density (ē.Å^−3^)	4.41/−1.77	1.02/−0.91

**Table 4:** Selected bond lengths (in Å) and angles (in deg.) in [RuT0B0(NO)]^3+^, and [RuT1B0(DMSO_(S)_)]^2+^.

	[RuT0B0(NO)]^3+^		[RuT1B0(DMSO_(S)_)]^2+^
	Complex 1 (Ru1)	Complex 2 (Ru2)	
*Ru-NO*			
Ru-N	1.766(6)	1.770(7)	
N-O	1.119(8)	1.135(9)	
Ru-N-O	177.7(6)	175.3(6)	
*Ru-DMSO*			
Ru-S			2.2666(4)
S-O			1.4826(12)
*Ru-tpy*			
Ru-N_central_	1.998(6)	1.996(6)	1.9735(12)
Ru-N_lateral_	2.093(6)	2.064(7)	2.0738(12)
	2.101(6)	2.084(7)	2.0842(12)
*Ru-bpy*			
Ru-N_transNO_	2.082(6)	2.092(7)	
Ru-N_transDMSO_			2.0912(12)
Ru-N	2.089(6)	2.077(6)	2.1037(12)

**Table 5:** Comparison of the Ru-DMSO_(O)_/Ru-DMSO_(S)_ ratios determined by ^1^H-NMR spectroscopy after one hour of irradiation of ruthenium complexes with various substituted terpyridine ligands at λ = 365 nm.

Complex	Substituent on Terpyridine	Ru-DMSO_(O)_/Ru-DMSO_(S)_		Computed *ε*_365_ (L mol^−1^ cm^−1^)
		Experimental ^1^	Computed ^2^	
[RuT0B0(DMSO(S))]^2+^	H	42%	53%	6021
[RuT0B0(DMSO(O))]^2+^	H			5317
[RuT1B0(DMSO(S))]^2+^	MeO-phenyl	25%	32%	9806
[RuT1B0(DMSO(O))]^2+^	MeO-phenyl			21,112
[RuT2B0(DMSO(S))]^2+^	Br-phenyl	31%	47%	12,365
[RuT2B0(DMSO(S))]^2+^	Br-phenyl			14,186
[RuT3B0(DMSO(S))]^2+^	NO_2_-phenyl	46%	64%	14,034
[RuT3B0(DMSO(S))]^2+^	NO_2_-phenyl			7794

^1^ determined by ^1^H-NMR spectroscopy under irradiation. ^2^ determined by the ratio of the extinction coefficient computed at λ = 365 nm.

**Table 6:** Computed absorption maxima (*λ*_max_ in nm), oscillator strength (*f*), composition and main character of the low-energy transition of the Ru-DMSO_(S)_ and Ru-DMSO_(O)_ complexes, compared with the experimental data available.

Compound	UV-vis		DFT Computed Data					
	*λ*_max_	*ε*	Transition *^a^*	*λ*	*f*	*λ*_max_ *^b^*	Composition *^c^*	Character *^d^*
[RuT0B0(DMSO_(S)_)]^2+^	419	6680	S_0_ → S_6_	395	0.104	395	0.53 χ_129__→ 132_ + 0.41 χ_129__→ 133_	Ru(d) → π(bpy/tpy)
[RuT0B0(DMSO_(O)_)]^2+^			S_0_ → S_6_	462	0.103	456	0.38 χ_128__→ 133_ – 0.32 χ_129__→ 1313_	Ru(d) → π(tpy)
[RuT1B0(DMSO_(S)_)]^2+^	436	6680	S_0_ → S_3_	440	0.311	430	0.61 χ_158__→ 159_	π(PhOMe)Ru(d) →π(tpy)
[RuT1B0(DMSO_(O)_)]^2+^			S_0_ → S_6_	474	0.134	472	0.40 χ_158__→ 161_ + 0.35 χ_158__→ 160_	π(PhOMe)Ru(d) → π(tpy)
[RuT2B0(DMSO_(S)_)]^2+^	433	11150	S_0_ → S_4_	427	0.159	409	0.53 χ_167__→ 170_ – 0.42 χ_165__→ 168_	Ru(d) → π(bpy)
[RuT2B0(DMSO_(O)_)]^2+^			S_0_ → S_6_	470	0.154	466	0.35 χ_167__→ 169_ – 0.34 χ_166__→ 168_	Ru(d) → π(tpy)
[RuT3B0(DMSO_(S)_)]^2+^	438	14500	S_0_ → S_4_	432	0.210	412	0.56 χ_159__→ 162_	Ru(d) → π(tpyPhNO_2_)
[RuT3B0(DMSO_(O)_)]^2+^			S_0_ → S_5_	477	0.181	478	0.34 χ_161__→ 164_ + 0.32 χ_161__→ 163_	Ru(d) → π(tpyPhNO_2_)

*^a^* Main transition bearing the first absorption band. *^b^* Computed absorption maximum of the first absorption band of the simulated spectra (see Supplementary Information). *^c^* Main excitations involved in the electronic transition. *^d^* Main character deduced from the natural transition orbitals (shown in Supplementary Information).

## Supplementary Materials

The following are available online: Figures S1 and S2: NMR Spectra and assignments for forms A, B, C; Figure S3: NMR Spectra forms C/D; Figure S4: Computed structure; Figure S5: NMR tracking for the irradiation of [RuT1B0(NO)](PF_6_)_3_ in DMSO; Figure S6: Simulated electronic spectra for the Ru-DMSO_(S)_ and Ru-DMSO_(O)_ complexes.
