# Supplementary material for: Multistep Photochemical Reactions of Polypyridine-Based Ruthenium Nitrosyl Complexes in Dimethylsulfoxide
Source: Molecules. 2020 May 8;25(9):2205. doi: 10.3390/molecules25092205 (PMC7248738; doi:10.3390/molecules25092205)

molecules

MDPI

Supporting Informations

Multistep photochemical reaction of polypyridine-based ruthenium nitrosyl complexes in dimethylsulfoxide

Natacha Marchenko, Pascal G. Lacroix, Valerii Bukhanko, Marine Tassé, Sonia Mallet-Ladeira, Martial Boggio-Pasqua and Isabelle Malfant

1. Assignment of ^{1}H-NMR signals for [RuT0B0(NO)]$(PF_6)_3$ (in DMSO)

2. Assignment of ^{1}H-NMR signals for [RuT1B0(NO)]$(PF_6)_3$ (in DMSO)

3.1. H-NMR ((400 MHz, DMSO-d6) of form D, obtained from a C/D mixture after irradiation of form C in DMSO, at = 365 nm.

4. Computed structures of $[RuT1B0(NO)]^{3+}$ and $[RuT1B0(DMSO)]^{2+}$

5. Relative concentration of [RuT1B0(DMSO$_{(S)}$)]$(PF_6)_2$ (form C) by NMR tracking

6. Simulated electronic spectra

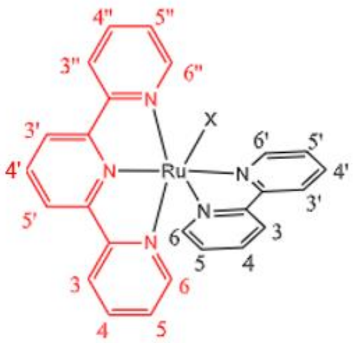

Figure 1. Assignment of ^{1}H-NMR signals for $[RuT0B0(NO)](PF_6)_3$ (in DMSO).

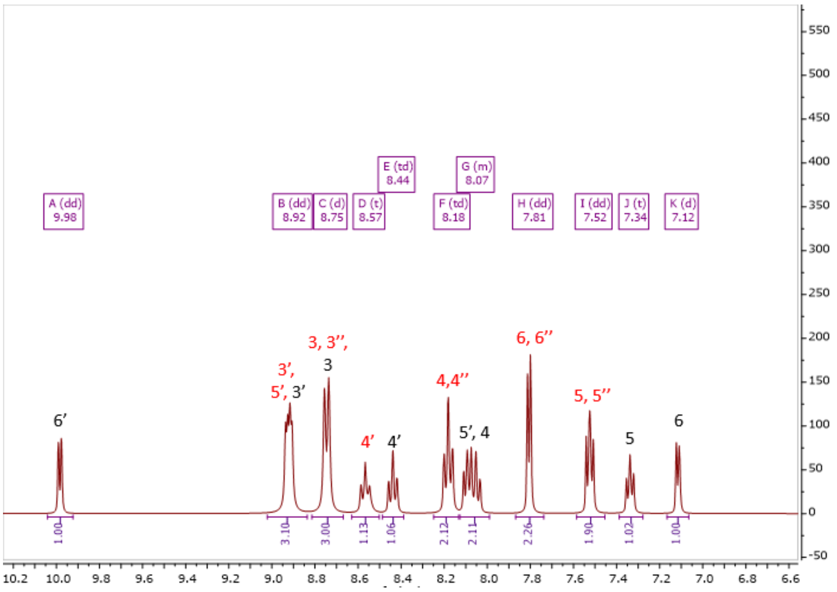

Form A.

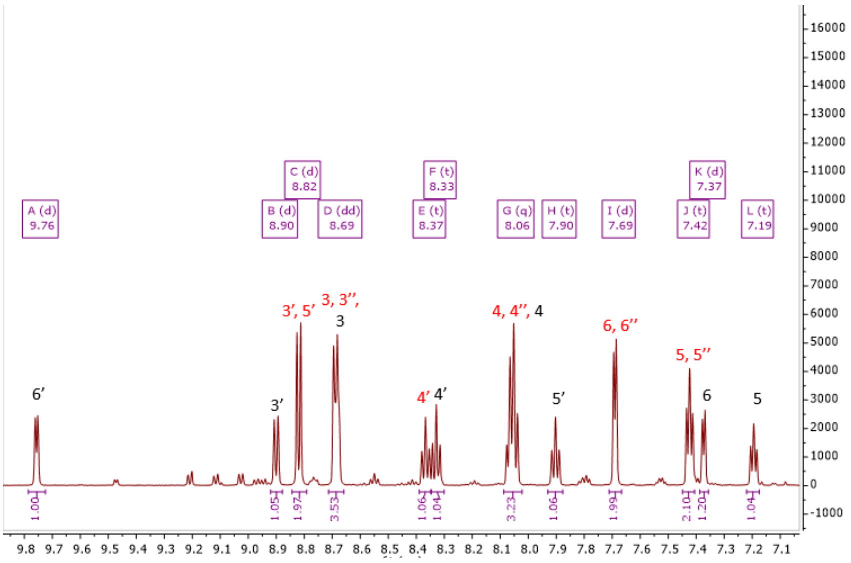

Form B.

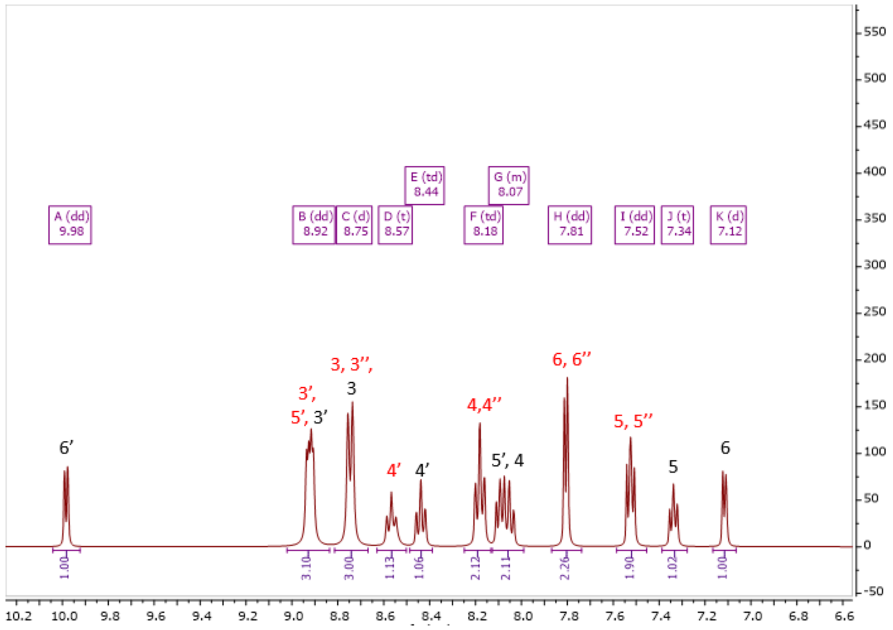
A (dd) 9.98
B (dd) 8.92
C (d) 8.75
D (t) 8.57
E (td) 8.44
F (td) 8.18
G (m) 8.07
H (dd) 7.81
I (dd) 7.52
J (t) 7.34
K (d) 7.12
6'
3', 5', 3'
3, 3'', 3
4'
4'
4,4''
5', 4
6, 6''
5, 5''
5
6
1.00
3.10
3.00
1.13
1.06
2.12
2.11
2.26
1.90
1.02
1.00

Form C.

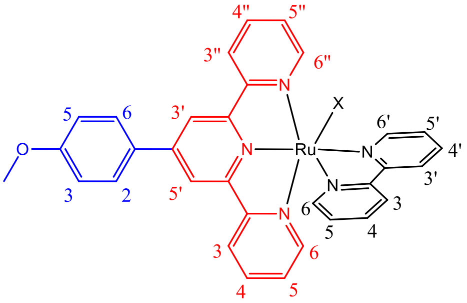

Figure 2. Assignment of ^{1}H-NMR signals for $[RuT1B0(NO)](PF_6)_3$ (in DMSO).

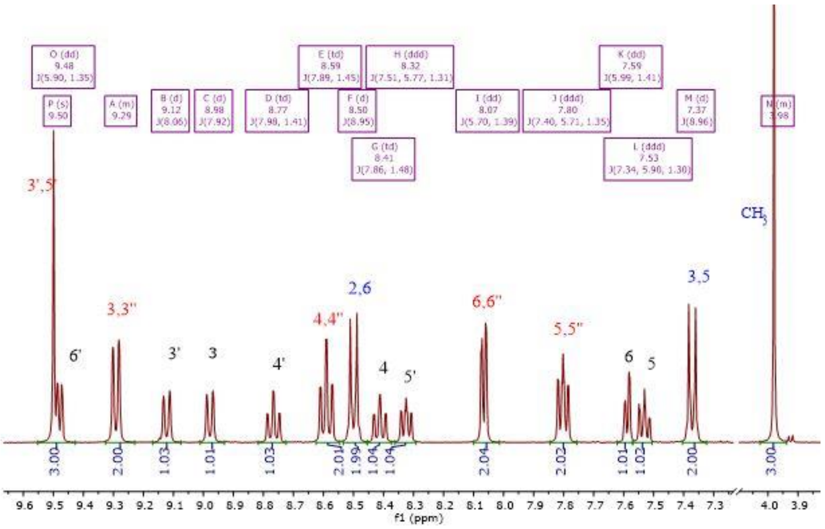

Form A.

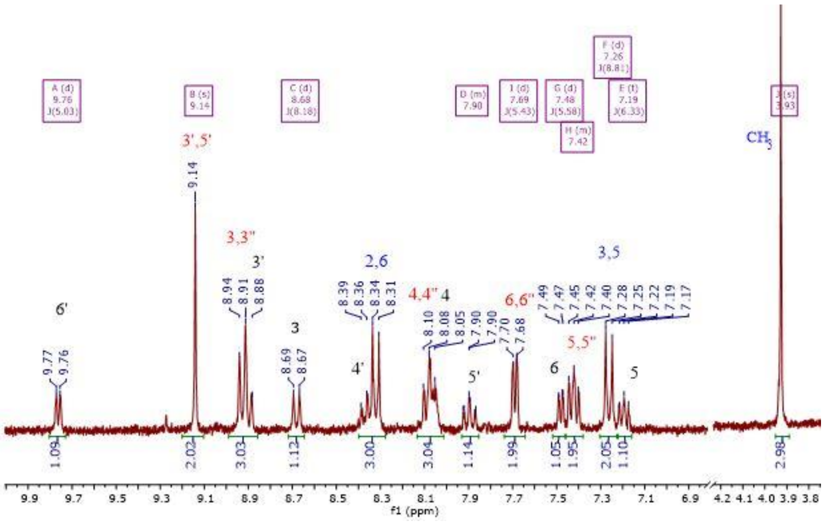

Form B.

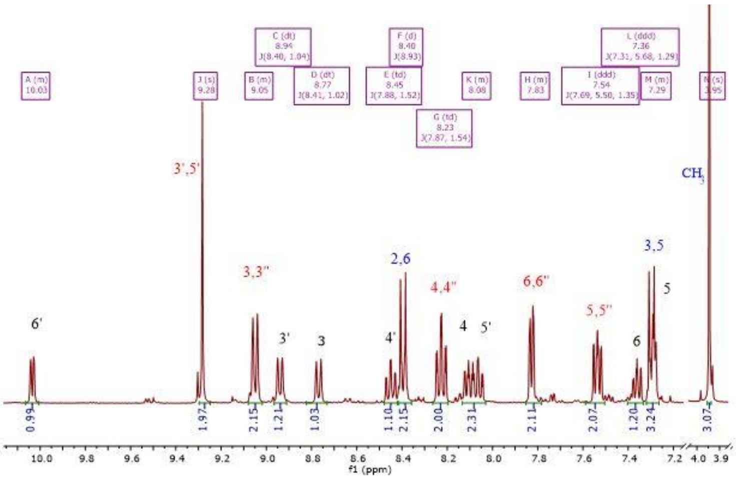
A (m) 10.03
J (s) 9.28
B (m) 9.05
C (dt) 8.94 J(8.40, 1.04)
D (dt) 8.77 J(8.41, 1.02)
E (td) 8.45 J(7.88, 1.52)
F (d) 8.40 J(8.93)
G (td) 8.23 J(7.87, 1.54)
K (m) 8.08
H (m) 7.83
I (ddd) 7.54 J(7.69, 5.50, 1.35)
L (ddd) 7.36 J(7.31, 5.68, 1.29)
M (m) 7.29
N (s) 3.95
6'
3',5'
3,3''
3'
3
4'
2,6
4,4''
4
5'
6,6''
5,5''
6
3,5
5
CH3
0.99
1.97
2.15
1.21
1.03
1.10
2.15
2.00
2.31
2.11
2.07
1.20
3.24
3.07
f1 (ppm)

Form C.

Figure 3. ^{1}H-NMR ((400 MHz, DMSO-d6) of form D, obtained from a C/D mixture after irradiation of form C in DMSO, at = 365 nm.

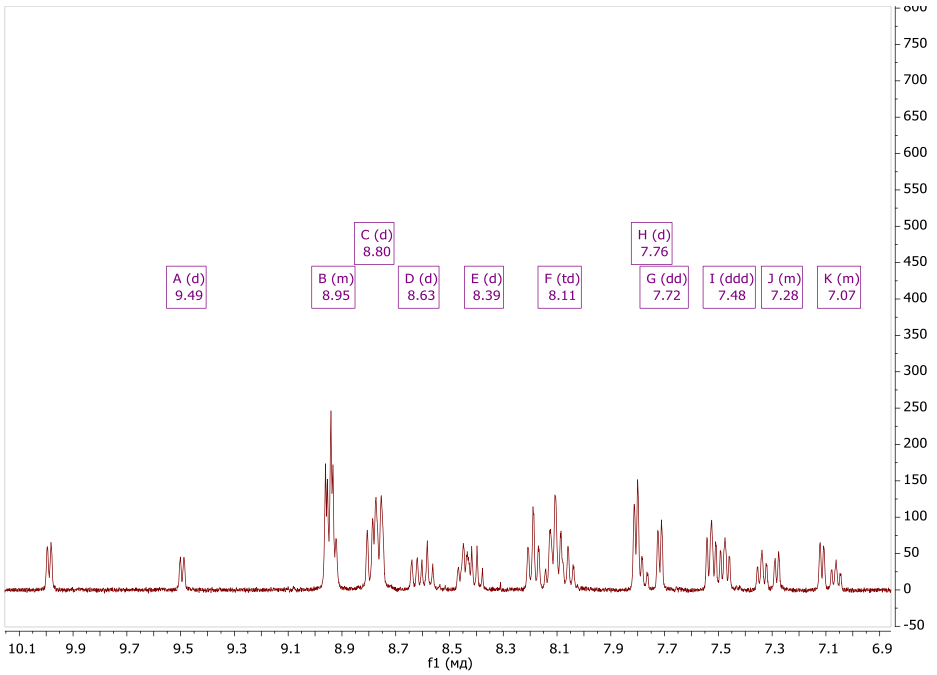

[RuT0B0(DMSO)(S)](PF6)2.

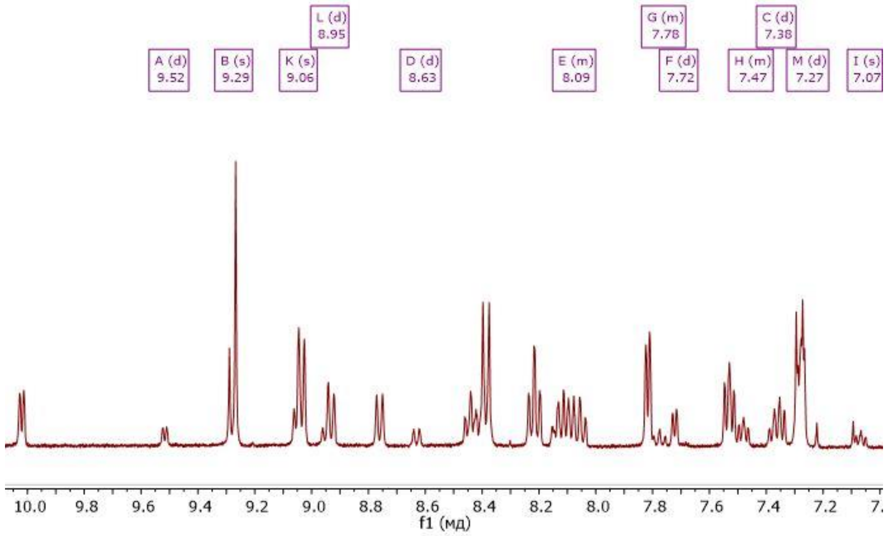

[RuT1B0(DMSO)(S)](PF6)2.

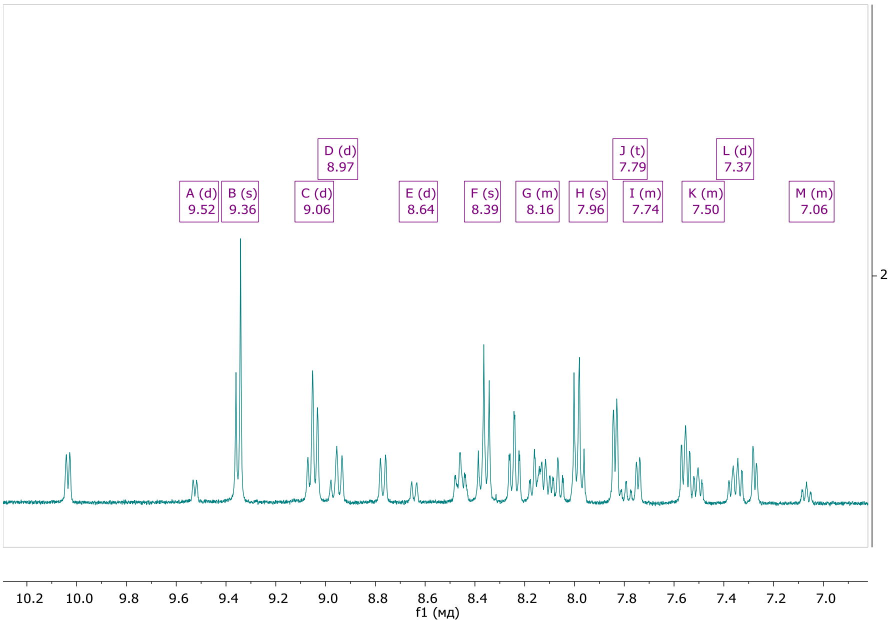

[RuT2B0(DMSO)(S)](PF$_6$)$_2$.

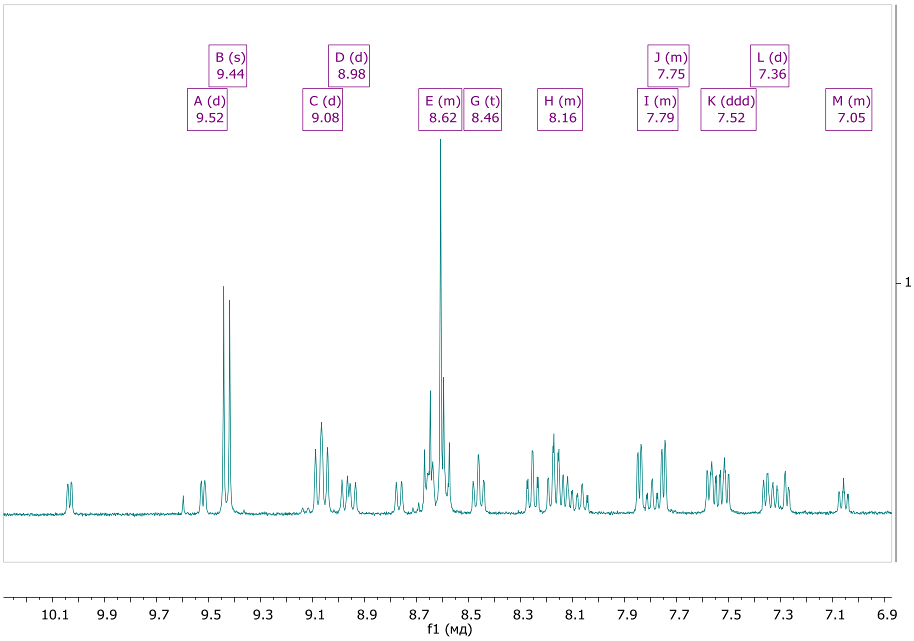

[RuT3B0(DMSO)(S)](PF$_6$)$_2$.

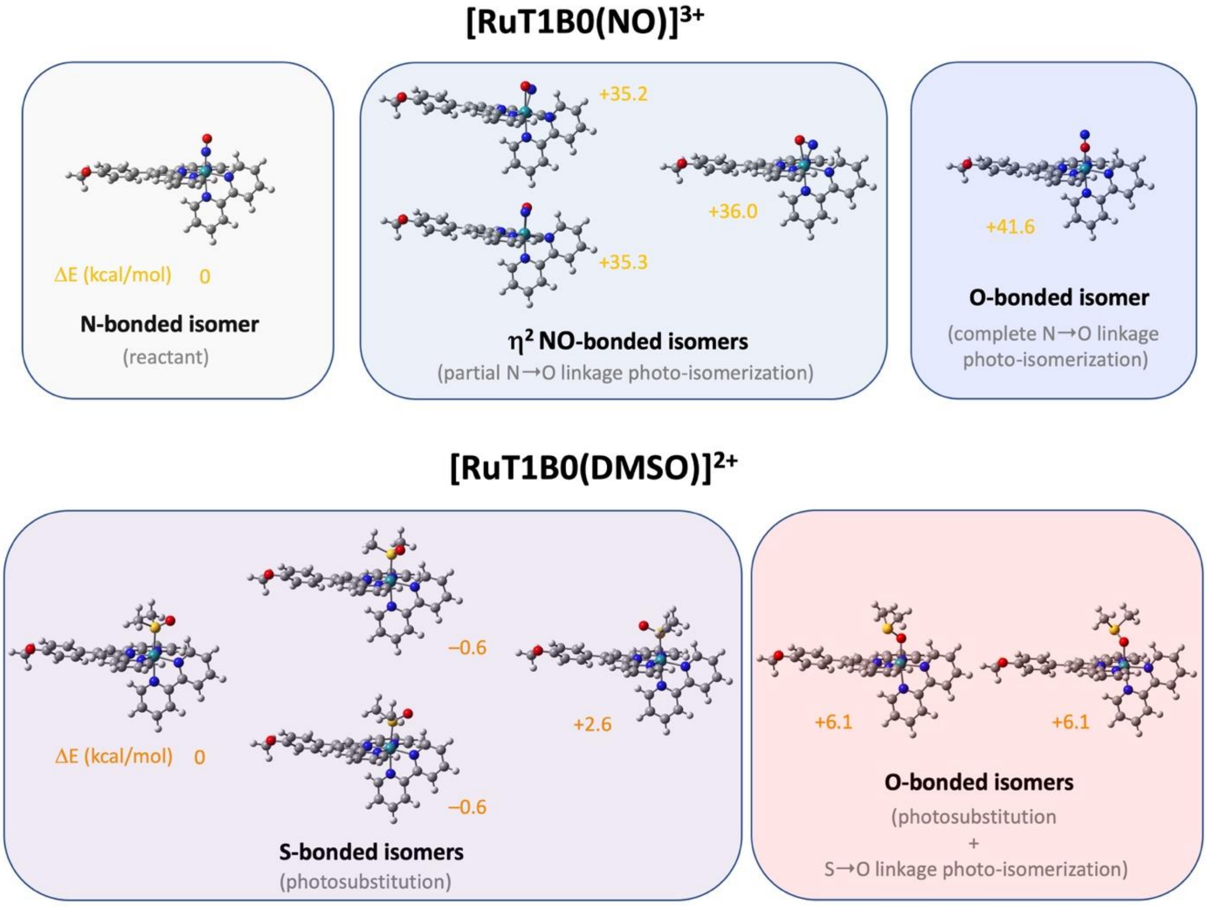

Figure 4. Computed structures of $[RuT1B0(NO)]^{3+}$ and $[RuT1B0(DMSO)]^{2+}$ complexes at the DFT level of theory and relative energies (in kcal/mol) of the different isomers.

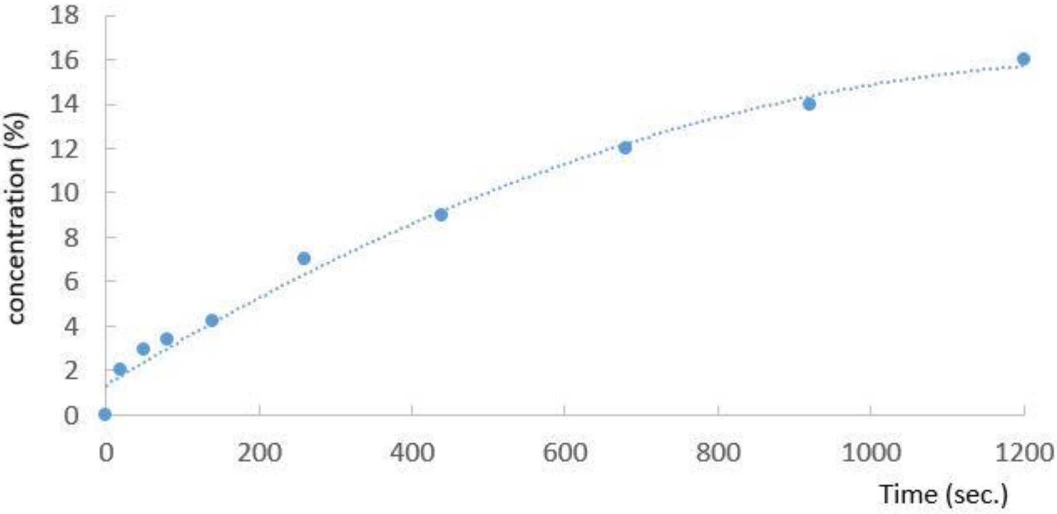

Figure 5. Relative concentration of [RuT1B0(DMSO$_{(S)}$)](PF$_6$)$_2$ (form C) from irradiation of [RuT1B0(NO)](PF$_6$)$_3$ (form A) in DMSO, estimated from ^{1}H-NMR tracking.

Figure 6. Simulated absorption spectra of S- and O-bonded [RuT0B0(DMSO)]$^{2+}$, [RuT1B0(DMSO)]$^{2+}$, [RuT2B0(DMSO)]$^{2+}$and [RuT3B0(DMSO)]$^{2+}$ complexes at the TD-DFT level of theory. Analysis of the lowest energy absorption band is given in terms of natural transition orbitals.

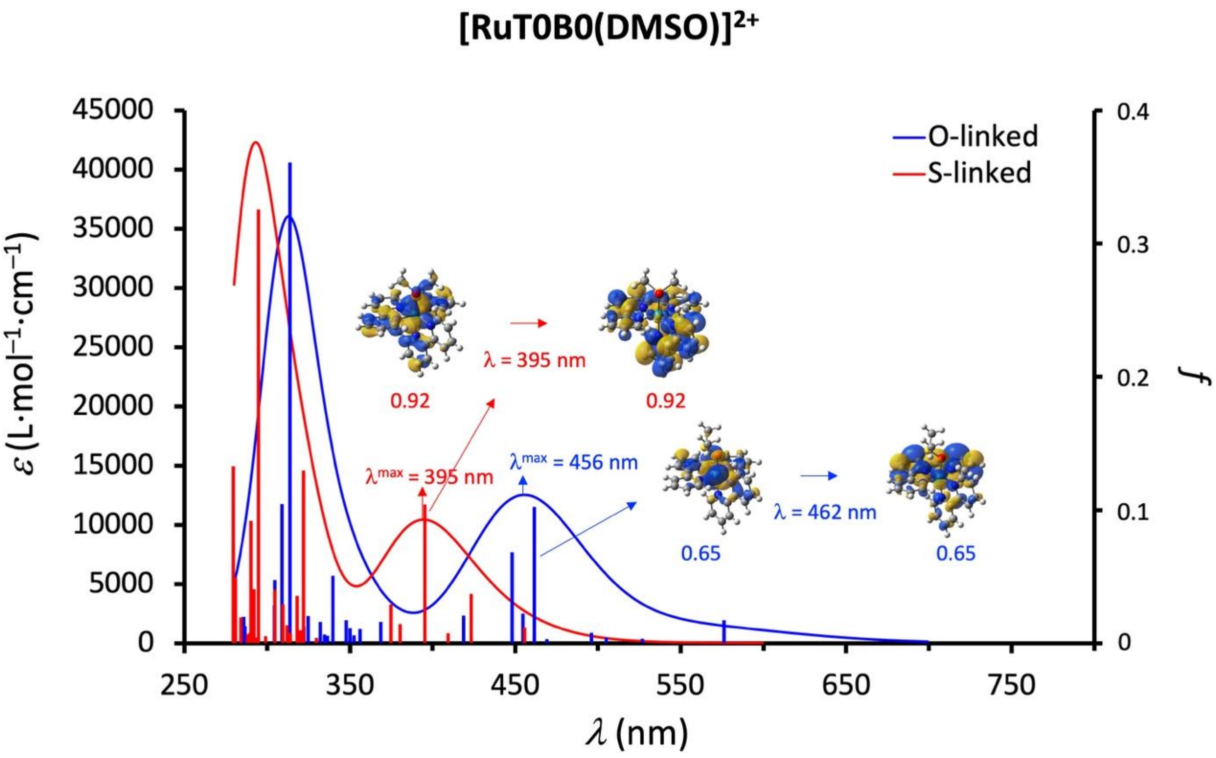

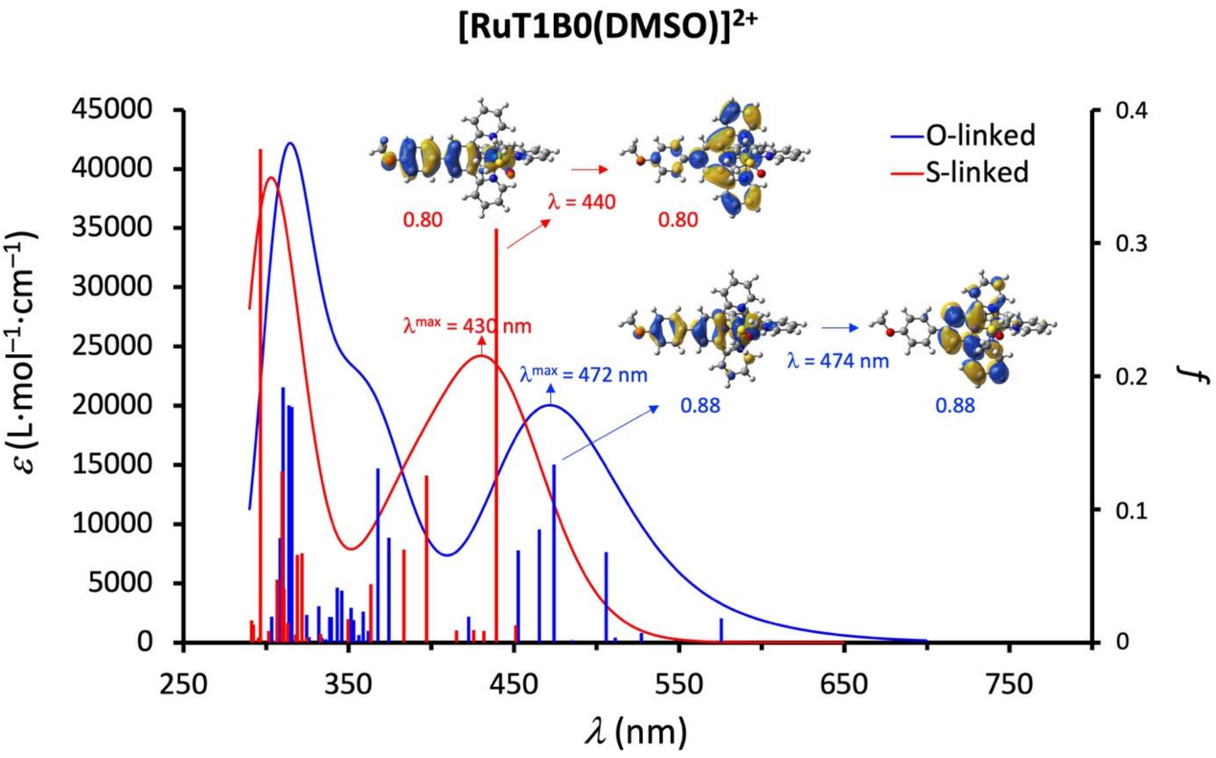

[RuT2B0(DMSO)]$^{2+}$

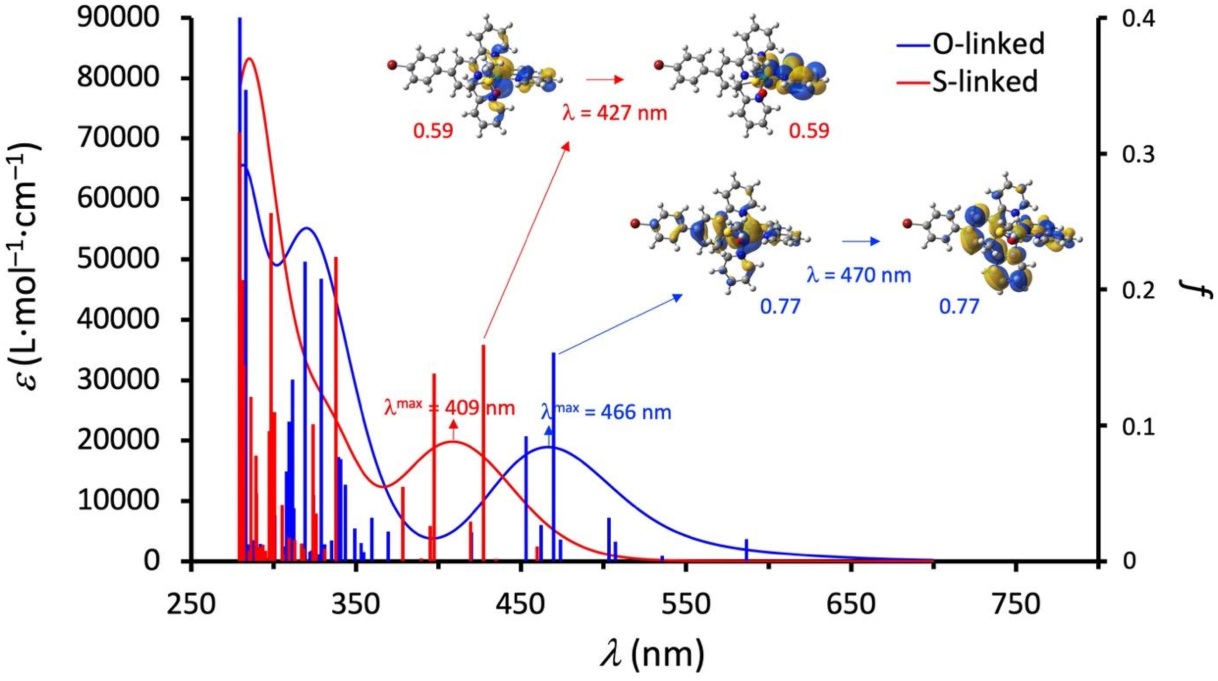

[RuT3B0(DMSO)]$^{2+}$

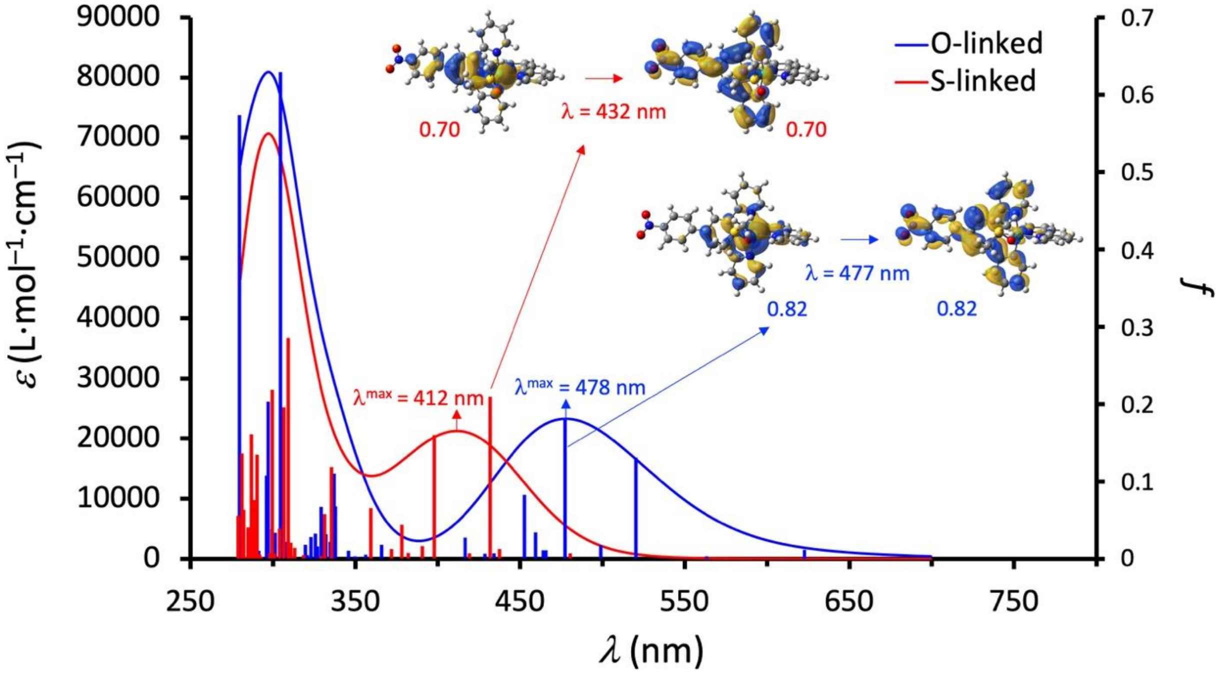

Supplement: Supplementary file 1 [file molecules-25-02205-s001.pdf]
